# Acquired resistance to KRAS G12C small-molecule inhibitors via genetic/nongenetic mechanisms in lung cancer

**DOI:** 10.1126/sciadv.ade3816

**Published:** 2023-10-13

**Authors:** Atish Mohanty, Arin Nam, Saumya Srivastava, Jeff Jones, Brett Lomenick, Sharad S. Singhal, Linlin Guo, Hyejin Cho, Aimin Li, Amita Behal, Tamara Mirzapoiazova, Erminia Massarelli, Marianna Koczywas, Leonidas D. Arvanitis, Tonya Walser, Victoria Villaflor, Stanley Hamilton, Isa Mambetsariev, Martin Sattler, Mohd W. Nasser, Maneesh Jain, Surinder K. Batra, Raffaella Soldi, Sunil Sharma, Marwan Fakih, Saswat Kumar Mohanty, Avijit Mainan, Xiwei Wu, Yihong Chen, Yanan He, Tsui-Fen Chou, Susmita Roy, John Orban, Prakash Kulkarni, Ravi Salgia

**Affiliations:** ^1^ Department of Medical Oncology and Experimental Therapeutics, City of Hope National Medical Center, Duarte, CA 91010, USA.; ^2^ Proteome Exploration Laboratory, California Institute of Technology, Pasadena, CA 91125, USA.; ^3^ Integrative Genomics Core, Beckman Research Institute, City of Hope, Monrovia, CA 91016, USA.; ^4^ Department of Pathology, City of Hope National Medical Center, Duarte, CA 91010,USA.; ^5^ Department of Medical Oncology, Dana-Farber Cancer Institute, Boston, MA 02215, USA.; ^6^ Biochemistry and Molecular Biology, University of Nebraska Medical Center, Omaha, NE 68198, USA.; ^7^ Applied Cancer Research and Drug Discovery Division, Translational Genomics Research Institute (TGen) of City of Hope, Phoenix, AZ 850043, USA.; ^8^ Department of Chemical Sciences, Indian Institute of Science Education and Research Kolkata, Mohanpur, West Bengal 741246, India.; ^9^ W. M. Keck Laboratory for Structural Biology, University of Maryland Institute for Bioscience and Biotechnology Research, Rockville, MD 20850, USA.; ^10^ Department of Chemistry and Biochemistry, University of Maryland, College Park, MD 20742, USA.

## Abstract

Inherent or acquired resistance to sotorasib poses a substantialt challenge for NSCLC treatment. Here, we demonstrate that acquired resistance to sotorasib in isogenic cells correlated with increased expression of integrin β4 (ITGB4), a component of the focal adhesion complex. Silencing ITGB4 in tolerant cells improved sotorasib sensitivity, while overexpressing ITGB4 enhanced tolerance to sotorasib by supporting AKT-mTOR bypass signaling. Chronic treatment with sotorasib induced WNT expression and activated the WNT/β-catenin signaling pathway. Thus, silencing both ITGB4 and β-catenin significantly improved sotorasib sensitivity in tolerant, acquired, and inherently resistant cells. In addition, the proteasome inhibitor carfilzomib (CFZ) exhibited synergism with sotorasib by down-regulating ITGB4 and β-catenin expression. Furthermore, adagrasib phenocopies the combination effect of sotorasib and CFZ by suppressing KRAS activity and inhibiting cell cycle progression in inherently resistant cells. Overall, our findings unveil previously unrecognized nongenetic mechanisms underlying resistance to sotorasib and propose a promising treatment strategy to overcome resistance.

## Teaser

Resistance to sotorasib in KRAS G12C lung cancer is due to genetic/non-genetic mechanisms and can be alleviated by carfilzomib.

## INTRODUCTION

Lung adenocarcinoma (LAUD) contributes to approximately 40% of all non–small cell lung cancer (NSCLC) cases (*1*). KRAS mutations are the most common gain-of-function alterations, accounting for approximately 30% of all lung adenocarcinomas (*1*–*4*). Most of the mutations affect codon 12, whereas the remainder typically affects codons 13 and 61 (*2*). Functionally, these mutations result in amino acid substitutions that impair K-ras p21 protein (KRAS) guanosine triphosphatase (GTPase) activity and render the oncoprotein constitutively active. Different amino acid substitutions induce distinct biological behaviors such as affecting patient prognosis and response to targeted therapies or chemotherapy (*5*–*10*).

Recently, sotorasib and adagrasib were developed as covalent inhibitors against KRAS G12C that interact with the mutant cysteine residue and lock the molecule in the guanosine diphosphate (GDP)–bound inactive state (*11*–*14*). However, progression-free survival with sotorasib was only 6.3 months, and only 45% of patients showed partial response to adagrasib (*14*). These partial responders often develop resistance. Drug resistance is generally thought to occur via genetic alterations that are irreversible (*15*–*21*). The term “resistance” includes both inherent resistance and acquired resistance. Inherent resistance refers to genetic changes that prevent a patient from responding to therapy altogether (*15*). On the other hand, acquired resistance is mediated through a reversible tolerant state. Tolerance occurs when a tumor initially responds to a drug but eventually becomes unresponsive due to nongenetic mechanisms (*15*). This reversible phenotype allows the cells to revert to their original state and repopulate, leading to tumor proliferation in the absence of the drug. In clinical terms, the tolerant state is analogous to stable diseases in patients. However, prolonged exposure to drugs by tolerant cells can result in the acquisition of mutations, leading to the development of irreversible resistance. While the genetic basis of drug resistance is well appreciated, the nongenetic mechanism leading to a tolerant state and lastly acquired resistance remains relatively poorly understood.

Several studies have highlighted the role of intrinsically disordered proteins (IDPs) in actuating nongenetic mechanisms that eventually lead to irreversible drug-resistant phenotype (*22*–*25*). Consistent with these observations, we showed that integrin β4 (ITGB4) and paxillin (PXN), key components of the focal adhesion complex, are IDPs with significant disordered regions and can induce cisplatin resistance in KRAS-mutant NSCLC through nongenetic mechanisms (*26*, *27*). Coexpression of these proteins correlated with poor patient survival, and perturbation of their signaling using the Food and Drug Administration–approved proteasome inhibitor, carfilzomib (CFZ), led to cell growth inhibition and sensitization to cisplatin (*26*). However, the contribution of these two proteins in acquiring tolerance or resistance against sotorasib is elusive. Further, the effect of CFZ in reverting sotorasib resistance is yet to be explored.

KRAS is a hybrid protein with several intrinsically disordered regions interdigitated between the highly ordered regions. Thus, any amino acid substitutions in the disordered region can induce conformational changes, which can alter its interaction with downstream signaling transducers, resulting in variable responses to therapy (*28*–*32*). Furthermore, it is also unclear whether failure to respond to sotorasib also results in loss of adagrasib sensitivity because both the molecules bind and inhibit KRAS G12C in its GDP-bound state. In this study, we examined the significance of the ITGB4 and Wnt/β-catenin signaling in acquiring tolerance to sotorasib and also determined the role of small-molecule inhibitor CFZ in reverting drug-tolerant phenotypes.

## RESULTS

### Sotorasib treatment affects ITGB4/PXN expression in sensitive cell lines

**Fig. 1.**
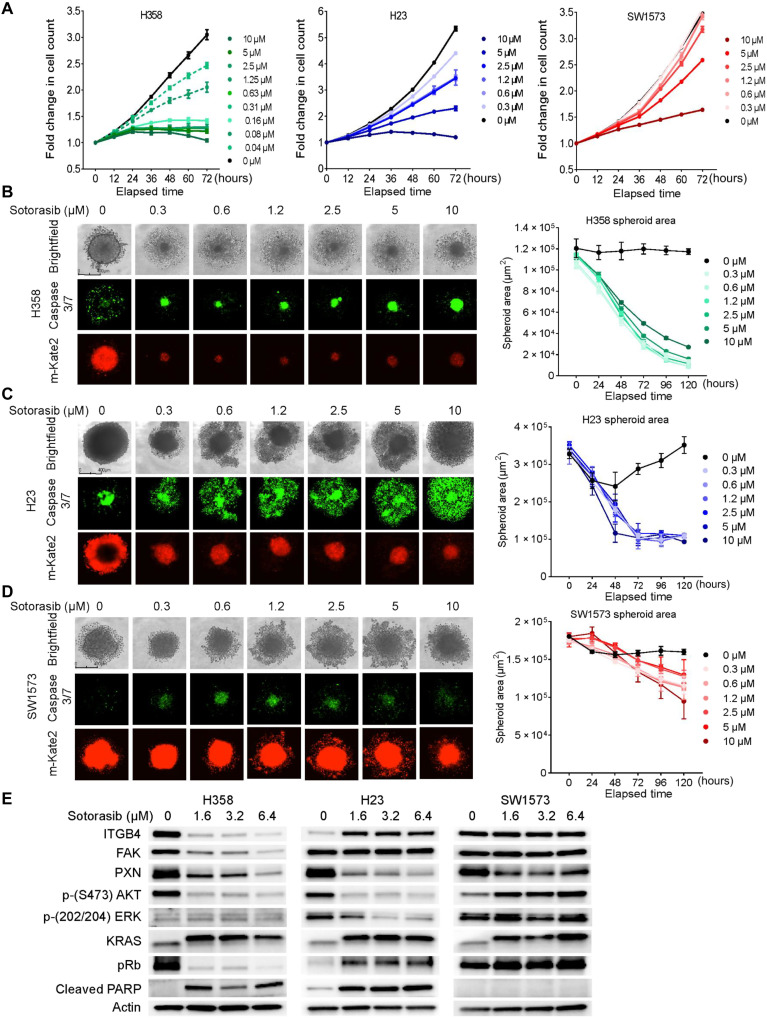
NSCLC KRAS G12C cell lines respond to sotorasib at varying concentrations. (**A**) NSCLC cell lines (H358, H23, and SW1573) with a KRAS G12C mutation were treated with an increasing concentration (0.3 to 10 μM) of sotorasib, and fold change in cell count was determined throughout 72 hours. Two-way ANOVA was used to calculate the statistical significance for each time point and for each drug concentration. *n* = 3 per group. (**B**) H358 cell line–derived spheroids were treated with an increasing concentration (0.3 to 10 μM) of sotorasib, and images were taken with the IncuCyte Live Cell Imaging System on day 5. Red fluorescence indicates cell viability, and green fluorescence indicates caspase 3/7 activity. The kinetics of the spheroid area was captured and plotted as graphs beside the images. Ordinary one-way analysis of variance (ANOVA) was used to calculate the statistical significance for each time point and each drug concentration. *n* = 4 per group. (**C** and **D**) Effect of increasing concentration of sotorasib on the H23- and SW1573-derived spheroids. Ordinary one-way ANOVA was used to calculate the statistical significance for each time point and for each drug concentration. *n* = 4 per group. (**E**) Immunoblot showing changes in the expression of KRAS and downstream signaling molecules upon sotorasib treatment (1.6, 3.2, and 6.4 μM).

We screened a panel of KRAS-G12C NSCLC cell lines and identified the ones that are inherently resistant or sensitive to sotorasib. We selected three cell lines, namely, H358, H23, and SW1573, based on their sensitivity to sotorasib in two-dimensional (2D) culture for further evaluation (Fig. 1A). The half-maximal inhibitory concentrations (IC_50_) for H358, H23, and SW1573 cells, after 72 hours of drug treatment, were determined to be 0.13, 3.2, and 9.6 μM, respectively (fig. S1A). On the basis of the IC_50_ values, we designated H358, H23, and SW1573 as sensitive, tolerant, and inherently resistant cell lines, respectively.

We also tested their sensitivity to sotorasib in 3D culture and observed significant inhibition in the spheroid area and an increase in apoptosis for H358 and H23 compared to SW1573 spheroids (Fig. 1, B to D, and fig. S1, B and C). Further, the three cell lines were treated with increasing concentrations of sotorasib for 72 hours, and changes in the downstream effectors were compared to the control. In H358 cells, a rapid decrease in ITGB4 expression was observed, whereas in H23 cells, it was up-regulated, and in SW1573 cells, it remained unchanged (Fig. 1E). PXN expression was inhibited in all three cell lines, whereas focal adhesion kinase (FAK) expression remained unchanged in H23 and SW1573 cells. Thus, the primary components of the focal adhesion complex ITGB4, PXN, and FAK showed a marked reduction in H358, which correlated with its sensitivity to sotorasib.

Furthermore, in the H358, the survival signaling through phosphorylated AKT was suppressed, and the apoptotic marker, cleaved PARP [poly (ADP-ribose) polymerase], was highly induced, which correlates with its sensitivity to sotorasib (Fig. 1E). Likewise, suppression of AKT phosphorylation and induction of cleaved PARP was observed in H23 cells (Fig. 1E). In contrast, AKT was hyperphosphorylated, and no activation of cleaved PARP was observed in SW1573 cells, which correlated with its resistant phenotype. Further, in H358 cells, the tumor suppressor retinoblastoma (Rb) was found to be hypo-phosphorylated, suggesting that it was active and inhibiting the cell cycle. Extracellular signal–regulated kinases (ERK) phosphorylation showed no detectable changes in the H358 and SW1573 cells compared to the H23 cells (Fig. 1E). Overall, the data from cell proliferation and immunoblotting support the categorization of the cell lines into sensitive, tolerant, and resistant phenotypes.

The cell lines were also tested using another KRAS inhibitor, ARS 1620, and the data lead to a similar conclusion as with sotorasib (fig. S2). These cell lines were treated with respective IC_50_ values of sotorasib or ARS1620, and changes in ITGB4/PXN expression and associated signaling showed a similar pattern for both the drugs across the three cell lines (fig. S3, A to C).

### Knocking down ITGB4 and PXN sensitizes cells to sotorasib

**Fig. 2.**
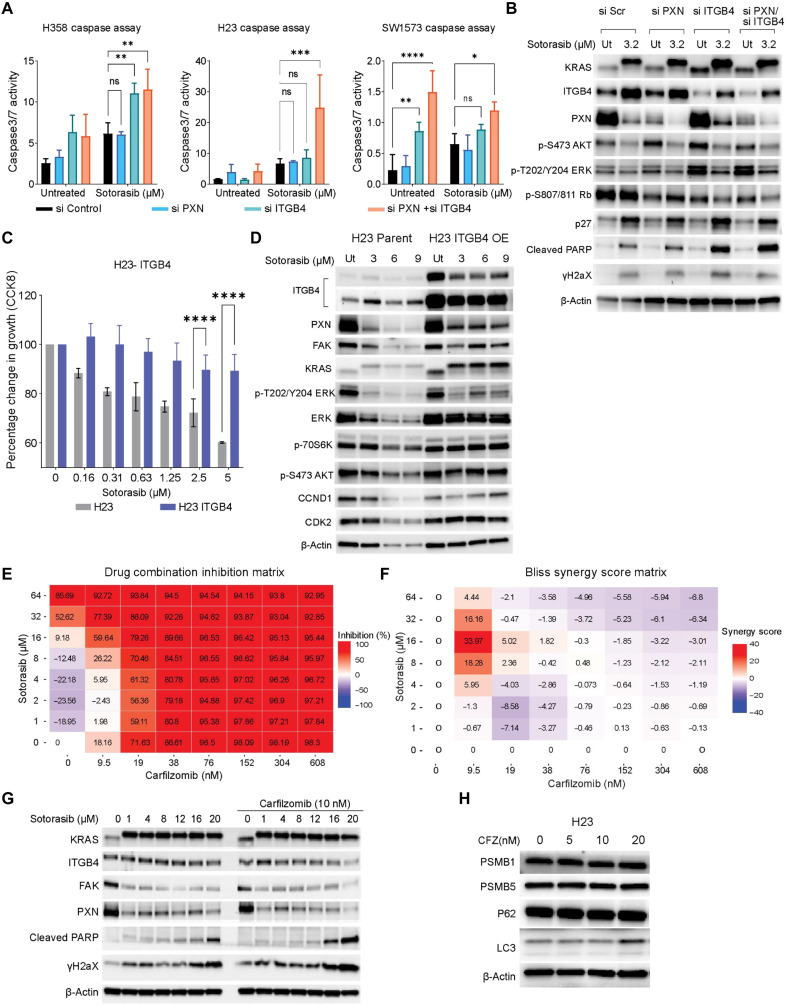
Inhibition of the ITGB4/PXN axis with siRNA or CFZ sensitizes cells to sotorasib treatment. (**A** and **B**) Effect of PXN and ITGB4 single knockdowns and double knockdown with sotorasib on caspase activity after 72 hours of drug treatment. Two-way ANOVA was used to calculate the statistical significance for each group (si Control, si PXN, si ITGB4, and si PXN + si ITGB4. *n* = 3 per group; ns, not significant; *P < 0.05, **P < 0.001, ***P = 0.0001, and ****P < 0.0001). (B) Immunoblot confirmed knockdown with ITGB4 and PXN siRNA, and effect of sotorasib on protein expression and signaling after 72 hours was determined. (**C**) H23 cells with ITGB4 overexpression (OE) were treated with an increasing concentration (0.3 to 5 μM) of sotorasib for 72 hours to determine the percentage change in cell growth. (**D**) H23 cells with ITGB4 overexpression were treated with an increasing concentration (3 to 9 μM) of sotorasib for 72 hours to determine the effect on protein expression and signaling with immunoblot. (**E** and **F**) SW1573 cells were treated with eight different concentrations of sotorasib and CFZ in the form of a matrix to determine the % inhibition of proliferation. Synergy scores were calculated and represented as a Bliss synergy matrix. (**G**) SW1573 cells were treated with an increasing concentration of sotorasib (1 to 20 μM) without or with the addition of CFZ (10 nM) for 72 hours to determine changes in protein expression and signaling by immunoblot. (**H**) Immunoblot showing changes in the component of proteasomal complex or autophagy-associated genes.

To investigate the role of ITGB4 and PXN in sotorasib tolerance, we used gene-specific small interfering RNA (siRNA) to silence ITGB4 or PXN, or both, in the three selected cell lines. The transfected cells were divided into two groups: untreated (control) and sotorasib treated. Caspase activity was then analyzed using the IncuCyte Live Cell Imaging System (Materials and Methods). In H358 and H23 cells, dual knockdown and sotorasib treatment led to an increase in caspase 3/7 activity, indicating enhanced apoptosis. However, in SW1573 cell lines, no significant change was observed (Fig. 2A). Knocking down ITGB4 alone or together with PXN reduced cell proliferation, and treatment with sotorasib further inhibited proliferation (fig. S3D). Knockdown of ITGB4 in addition to sotorasib treatment inhibited activation of AKT and ERK, reduced Rb phosphorylation, and increased p27 expression leading to inhibition of cell growth signaling and cell cycle progression. Moreover, there was an increase in the expression of phosphorylated H2A histone family member X (γH2AX) and cleaved PARP, indicating induction of DNA damage and apoptosis. Thus, targeting ITGB4 in KRAS G12C mutant cell lines exhibited an additive effect on the growth inhibition mediated by sotorasib (Fig. 2B).

### ITGB4 overexpression restrains sotorasib toxicity

To further validate the role of ITGB4 in sotorasib tolerance, we generated a H23 stable cell line overexpressing ITGB4. Parental and ITGB4-overexpressing cells were treated with increasing concentrations of sotorasib, and growth inhibition was determined after 72 hours of treatment. Cell growth was inhibited by 40% in parental cells, whereas 15% inhibition was observed in ITGB4-overexpressing cells upon sotorasib (5 μM) treatment (Fig. 2C). Next, to discern the effects of ITGB4 overexpression on downstream signaling, we treated the parental and ITGB4-overexpressing cells with 3, 6, and 9 μM sotorasib for 72 hours and performed immunoblotting analysis (Fig. 2D). PXN, FAK, cyclin D1 (CCND1), CDK2, β-actin, and total ERK expression were observed to be substantially down-regulated in the parental H23 cells but not in ITGB4-overexpressing cells. Furthermore, in ITGB4-overexpressing cells, AKT and p70S6 kinase phosphorylation remain unchanged, whereas the ERK phosphorylation was inhibited upon sotorasib treatment (Fig. 2D). Thus, the data suggested that ITGB4 overexpression in H23 cells promotes sotorasib tolerance by supporting AKT-mTOR (mamalian target of rapamycin) bypass signaling.

To confirm this correlation between ITGB4 and sotorasib, we generated isogenic resistant cells using the H23-tolerant cells. First, we cultured the H23 cells in the presence of sotorasib to make them resistant to 3.2 μM sotorasib, and eventually, we increased the drug concentration to make them resistant to 7.5 μM and, lastly, 20 μM sotorasib. In addition, we also exposed the H358 cells to IC_50_ or a higher concentration of sotorasib, but isogenic resistant cells could not be devloped. However, isogenic resistant SW1573 cells were generated by exposing these cells to IC_50_ concentration of sotorasib. Immunoblotting analysis revealed an increase in ITGB4 expression in isogenic resistant H23 and SW1573 cells compared to parental cells (fig. S3E). On the other hand, the ITGB4 expression in the H358 cells decreased, suggesting that cells with unstable ITGB4 may not develop tolerance to sotorasib. These findings again re-emphasize the significance of ITGB4 expression in overcoming the inhibitory effect of sotorasib.

### CFZ acts synergistically with sotorasib

In our previous study, CFZ was found to suppress ITGB4 expression and sensitize the KRAS G12A-mutant cell line to platinum therapy (*26*). Therefore, we investigated the combined effect of CFZ and sotorasib in 48 different combinations against the inherently resistant cell line SW1573. Individually, CFZ (9.5 nM) inhibited cell proliferation by 18%, and sotorasib (16 μM) inhibited proliferation by 9.18%. However, the combination of CFZ (9.5 nM) and sotorasib (16 μM) inhibited cell proliferation by approximately 60% (Fig. 2E). Using the SynergyFinder (Bliss, SynergyFinder web application and SynergyFinder 2.0: visual analytics of multidrug combination synergies) analysis tool, we identified that the drug combination of 9.5 nM CFZ and 16 μM sotorasib to be synergistic with a synergy score of 33.97 (Fig. 2F and fig. S4, A and B). Cell growth analysis also confirmed a statistically significant reduction in cell growth by drug combination, relative to a single drug alone in both normoxic and hypoxic conditions (fig. S4, C to E). A combination of 16 or 20 μM sotorasib and 10 nM CFZ reduced the expression of ITGB4, PXN, and FAK in SW1573 cells and simultaneously activated the apoptotic markers cleaved PARP and γH2AX (Fig. 2G). The result was recapitulated using the H23 cell line as well (fig. S4F). Because CFZ is a proteasome inhibitor, we determined whether CFZ could induce proteasomal inhibition at 10 nM concentration. We did not observe any notable changes in the components of the proteasomal complex proteins like Proteasomal Subnuit Beta1 (PSMB1), Proteasomal Subunit Beta5 (PSMB5), or autophagy-associated proteins p62 (Sequestome 1) and LC3 ( Microtubule associated protein 1 Light Chain 3 Alpha), indicating that the synergy was independent of proteasomal inhibition (Fig. 2H). Together, these results suggest that CFZ can potentially be used against sotorasib-refractory ITGB4-overexpressing KRAS G12C tumors.

### Wnt family member 2 expression up-regulated in response to chronic sotorasib treatment

**Fig. 3.**
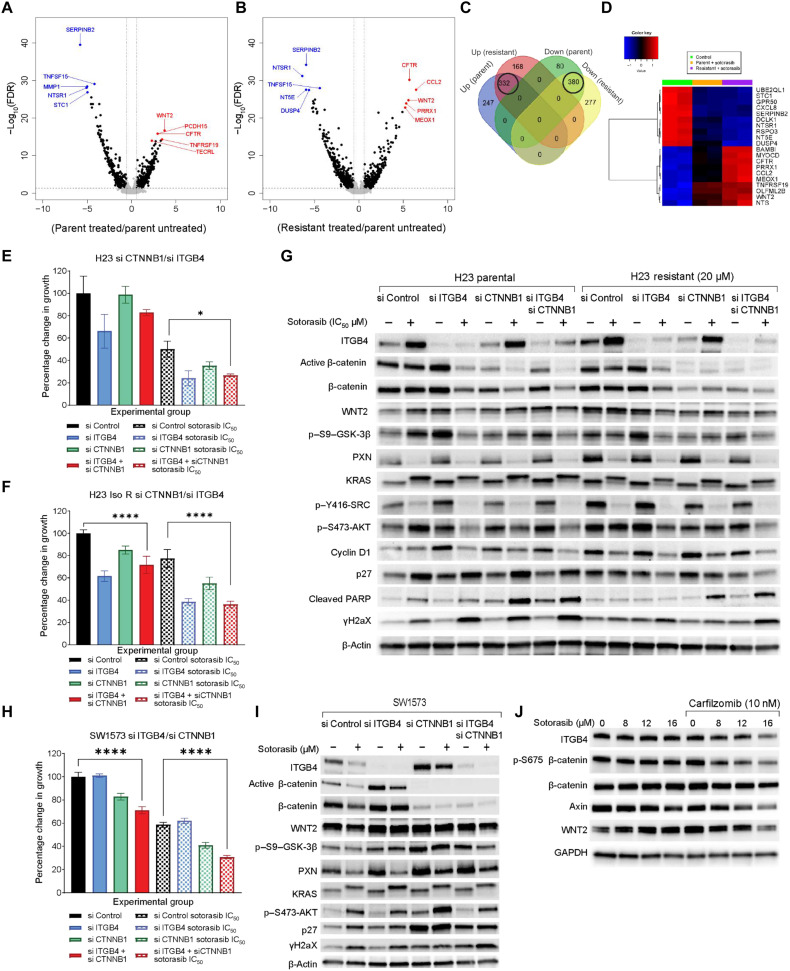
RNA sequencing reveals WNT2 up-regulation upon sotorasib treatment. Volcano plot representing gene expression changes between (**A**) H23 parental cells, sotorasib treated versus untreated, and (**B**) isogenic resistant H23 cells compared to parental cells. Statistical significance was calculated for control verse treatment. *n* = 3 per group. (**C**) Number of overlapping and unique genes that were up-regulated or down-regulated with respect to treatment represented as a Venn diagram. (**D**) Heatmap representing top 10 overlapping up-regulated and down-regulated genes based on average fold change. (**E** and **F**) Effect of 3.2 μM sotorasib on H23 parental or isogenic resistant (Iso R) cells having knockdown of ITGB4 or CTNNB1 or both represented as percent change in growth at 96 hours (bar graph), respectively. Two-way ANOVA was used to calculate the statistical significance for each time point and for each condition (si Control, si ITGB4, si CTNNB1, si CTNNB1 + si ITGB4; *n* = 3 per group; ****P < 0.0001. (**G**) Immunoblot confirmed knockdown of ITGB4 and β-catenin in H23 parental cells and H23 sotorasib (20 μM) resistant cells. These cells were also treated with 3.2 μM sotorasib for 72 hours to identify changes in protein expression and signaling. (**H**) Representing the effect of ITGB4 and CTNNB1 single knockdown or double knockdown together with 10 μM sotorasib as a percent change in growth. Two-way ANOVA was used to calculate the statistical significance for each time point and each condition (si Control, si PXN, si ITGB4, and si PXN + si ITGB4; *n* = 3 per group; ****P < 0.0001. (**I**) Immunoblot showing knockdown of ITGB4 and β-catenin and changes in expression of active β-catenin, γH2AX, and p27 in SW1573 cells. (**J**) Immunoblot showing the reduction in the expression of WNT2, ITGB4, phospho, and total β-catenin in the SW1573 treated with sotorasib and CFZ drug combination.

We performed a differential gene expression analysis to discern any additional mechanisms of acquired resistance to sotorasib. RNA was extracted from H23 parental cells after treating them with 3.2 μM (H23 IC_50_) of sotorasib for 3 days. Similarly, RNA was also extracted from isogenic resistant H23 cells that were able to tolerate and grow in 7.5 μM sotorasib for library preparation and sequencing. Global changes in gene expression in parental cells in response to sotorasib treatment (volcano plot, Fig. 3A) and changes in isogenic resistant cells compared to untreated parental cells were discerned (volcano plot, Fig. 3B). We identified 168 unique genes that were up-regulated in the resistant cells compared to 247 genes for the parental cells. Likewise, 277 unique genes were down-regulated in the resistant cells compared to the 80 genes for the parental cells (Fig. 3C). The differential expression analysis also revealed 332 up-regulated and 380 down-regulated genes that overlapped across both the treatments (Fig. 3C). The top 10 genes that were consistently up-regulated in both parental and resistant cells were *CCL2*, *CFTR*, *WNT2*, *PRRX1*, *MEOX1*, *MYOCD*, *BAMBI*, *COL26A1*, *CTTNBP2*, and *TNFRSF19*. The genes that consistently down-regulated were *UBE2QL1*, *DCLK1*, *SHH*, *CRH*, *RSPO3*, *STC1*, *DUSP4*, *NT5E*, *SERPINB2*, and *NTSR1* (Fig. 3D). Gene set enrichment analyses (GSEAs) were performed to identify up-regulated pathways contributing to sotorasib resistance. However, no significant changes were observed except for the inhibition of tumor necrosis factor–α (TNF-α) and KRAS signaling pathways (fig. S5, A to E, and table S1 to S4). The gene expression changes were further validated using quantitative polymerase chain reaction (qPCR) and immunoblot assays (fig. S5, F to H). Next, we performed exome sequencing analysis of H23 cells resistant to 7.5 μM sotorasib (Iso 7.5 cells) or 20 μM sotorasib (Iso 20 cells) to identify genes that could have contributed to developing acquired resistance. The analysis revealed nonsynonymous mutations in some genes, but there were no statistically significant changes in the expression of these mutated genes (fig. S5I).

Overall, the GSEA analysis did not reveal any notable activation of pathways that could contribute to drug resistance. Therefore, we sought to understand the contributions of the genes up-regulated upon sotorasib treatment. Because Wnt family member 2 (WNT2) was up-regulated in both the parental and isogenic resistant cell lines, and its downstream signaling through β-catenin is known to induce stemness and drug resistance in various solid tumor studies, we selected WNT2 for further investigation. We first generated a WNT2 knockout (KO) H23 cell line using the combination of WNT2 CRISPR-Cas9 KO and WNT2 homology directed repair (HDR) plasmids from Santa Cruz Biotechnologies. The KO cells showed statistically significant inhibition in cell growth upon sotorasib treatment, whereas the effect was insignificant for the WNT2 KO SW1573 cells (fig. S6, A and B). The sensitivity of WNT2 KO H23 to sotorasib further increased upon ITGB4 knockdown (fig. S6C). It is possible that SW1573 cells were insensitive to WNT2 knockdown due to the expression of a mutant form of CTNNB1, which remains constitutively active. Therefore, we evaluated the role of β-catenin in acquired resistance to sotorasib.

### Suppressing ITGB4 and β-catenin sensitizes resistant cells to sotorasib

We sought to compare the effect of sotorasib on the cells after knocking down β-catenin or ITGB4 or both. The H23 parental and their isogenic resistant cell lines were transfected with 10 nM CTNNB1 or ITGB4 or both siRNAs, and their proliferation in response to sotorasib treatment was determined (Fig. 3, E and F). Sotorasib was found to have a stronger inhibitory effect on the cells that were knocked down for both ITGB4 and β-catenin compared to control cells knocked down with scramble siRNA. Detailed cell proliferation kinetics concerning knockdown are shown in fig. S6 (D and E). Sotorasib treatment on ITGB4 knockdown cells decreased the expression of total and active β-catenin and increased the expression of p27, cleaved PARP, and γH2AX (Fig. 3G). The dual knockdown further added to this phenotype, as inferred from the more robust activation of p27, cleaved PARP, and γH2AX. This suggested that the inhibition of both genes synergizes with sotorasib to generate a stronger apoptotic phenotype (Fig. 3G). The lysates from isogenic sotorasib-resistant cells also confirmed a weaker apoptotic phenotype in the ITGB4 single knockdown setting and stronger in the dual knockdown setting (Fig. 3G).

We further evaluated the CTNNB1 and ITGB4 knockdown effect in the sotorasib-resistant SW1573 cells. As expected, sotorasib treatment more effectively inhibited the growth of cells with double knockdown, with 70% inhibition compared to 40% in parental cells (Fig. 3H and fig. S6F). In immunoblotting experiments, we observed an increase in the expression of ITGB4 in CTNNB1 knockdown lysates. Similarly, we observed a higher expression of β-catenin in ITGB4 knockdown lysates (Fig. 3I). These results suggested that up-regulation of either molecule is essential for overcoming KRAS inhibitor-induced drug toxicity. Therefore, suppressing the signaling induced by these two proteins could overcome sotorasib resistance in SW1573 cells.

To address this possibility, we treated SW1573 cells with sotorasib and CFZ for 72 hours and analyzed the expression of ITGB4 and β-catenin by immunoblotting. The results showed a reduction in ITGB4, Axin, WNT2, and β-catenin expression (Fig. 3J), further highlighting the effectiveness of CFZ and sotorasib in disrupting the signaling mediated by these proteins. As an alternative to CTNNB1 knockdown, we tested the combination of the β-catenin inhibitor BC2059 and sotorasib on SW1573 cells and found the combination to have an additive effect. However, the precise mechanism of action of BC2059 was uncertain as it was ineffective in down-regulating the expression of total or phospho–β-catenin (fig. S7, A to F).

### Sotorasib-resistant cells are sensitive to adagrasib, and adagrasib acts additively with CFZ

**Fig. 4.**
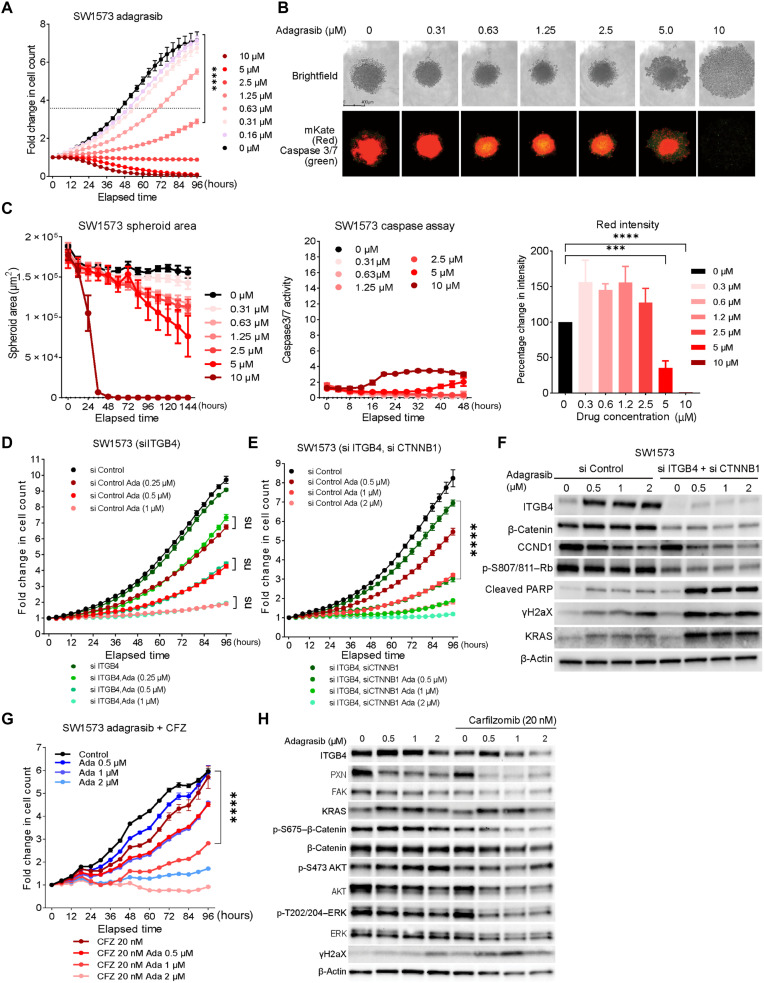
Adagrasib is effective in inhibiting sotorasib-resistant NSCLC cells. (**A**) Fold change in SW1573 cells growth with increasing concentration of adagrasib (0.6 to 10 μM). The dotted line represents a fold change in cell count, which corresponds to half of the total fold change shown by control cells. Statistical significance was calculated using two-way ANOVA for each time point and drug concentration. *n* = 3 per group; ****P < 0.0001. (**B**) IncuCyte images of SW1573 spheroids on day 5 of adagrasib (0.31 to 10 μM) treatment. (**C**) Changes in the spheroid area, caspase 3/7 activity, and red intensity. 10 μM adagrasib disintegrated the SW1573 spheroid and increased caspase 3/7 activity by threefold. Statistical significance was calculated using two-way ANOVA. *n* = 3 per group. (**D**) Live cell proliferation assay of ITGB4 siRNA-transfected SW1573 cells with and without adagrasib treatment for 96 hours. Two-way ANOVA was used to calculate the statistical significance (si Control or si ITGB4; *n* = 3 per group; ****P < 0.0001). (**E**) Effect of ITGB4/CTNNB1 double knockdown on SW1573 cells proliferation with and without adagrasib treatment for 96 hours (si Control, si ITGB4, Si CTNNB1, si CTNNB1 + si ITGB4; *n* = 3 per group; two-way ANOVA test, ****P < 0.0001). (**F**) ITGB4/CTNNB1double knockdown and adagrasib treatment induced expression of cleaved PARP, γH2AX expression, and inhibited Rb phosphorylation. (**G**) Inhibitory effect of adagrasib (0.5 to 2 μM) and CFZ (20 nM) on the SW1573 cell proliferation was additive. *n* = 3 per group. Two-way ANOVA test, ****P < 0.0001. (**H**) Immunoblot analysis of the signaling changes induced by adagrasib and CFZ combination. The combination treatment inhibited the expression of activated AKT, ERK, and β-catenin required for drug resistance.

Next, we asked whether sotorasib-resistant cells are also resistant to adagrasib. We used SW1573 cells and found adagrasib to be a more effective inhibitor compared to sotorasib (80% inhibition at 5 μM and 95% inhibition at 10 μM concentrations) (Fig. 4A). The IC_50_ concentration for adagrasib against SW1573 was calculated to be 4.13 μM, which was lower than that of sotorasib. Next, the efficacy of adagrasib in 3D spheroid cultures was assessed by measuring changes in spheroid growth, caspase activity, and red spheroid intensity (Fig. 4, B and C, and fig. S8, C to F). Spheroid growth was completely abrogated at 10 μM adagrasib, and at 5 μM, spheroid integrity was reduced. However, we observed no marked increase in caspase 3/7 activity (Fig. 4, B and C). Adagrasib was found to activate cleaved PARP and γH2AX expression in SW1573, which was not observed in sotorasib treatment, suggesting activation of alternative cell death pathways in these cells (fig. S8B). The IC_50_ concentrations for adagrasib for H358 and H23 cells were also found to be lower than that of sotorasib (fig. S8A). Thus, overall, the results indicated that sotorasib resistance does not confer resistance to adagrasib.

We repeated ITGB4 knockdown in the SW1573 cells and treated them with adagrasib but did not observe any statistically significant inhibition in cell growth (Fig. 4D, SW1573 si ITGB4 versus si ITGB4–Ada). Furthermore, we tested the inhibitory effects of adagrasib on cells with both CTNNB1 and ITGB4 knockdown. The cells were transfected with 10 nM respective siRNA and were treated with increasing concentrations of adagrasib to determine the changes in growth inhibition. No statistically significant difference was observed between the scrambled knockdown (eightfold, black line, Fig. 4E) and the double knockdown (sevenfold green line, Fig. 4E). However, 0.5 μM adagrasib significantly reduced the growth of double knockdown cells to threefold compared to fivefold growth for control cells (Fig. 4E). A stronger inhibition on the double knockdown cells was observed at a higher concentration of adagrasib (Fig. 4E). Immunoblotting analysis confirmed the knockdown of ITGB4 and β-catenin and in parallel revealed down-regulation of CCND1, phospho-Rb, and up-regulation of cleaved PARP and γH2AX upon adagrasib treatment (Fig. 4F). Together, these data reemphasized that targeting both ITGB4 and β-catenin can sensitize cells to adagrasib therapy even at a minimal concentration of 0.5 μM. We also analyzed the effect of adagrasib on WNT2 KO SW1573 and H23 cells and found the effect to be additive (fig. S8G).

Next, we evaluated the synergy between adagrasib and CFZ drug combinations and found the inhibitory effect to be additive (fig. S8H). We further explored the effect of this drug combination on cell proliferation. The cells treated with CFZ (20 nM) and adagrasib (1 μM) combination showed threefold increase, whereas a fivefold increase was observed by 1 μM adagrasib alone. At 2 μM concentration, adagrasib had a cytostatic effect, whereas the combination further suppressed the cell number, suggesting that the decrease in cell number was due to cell death (Fig. 4G). Immunoblotting analysis revealed that the combination can suppress the expression of ITGB4, phospho–β-catenin, phospho-AKT, and phospho-ERK effectively (Fig. 4H, immunoblots).

### Differential growth inhibition effect of sotorasib and adagrasib

**Fig. 5.**
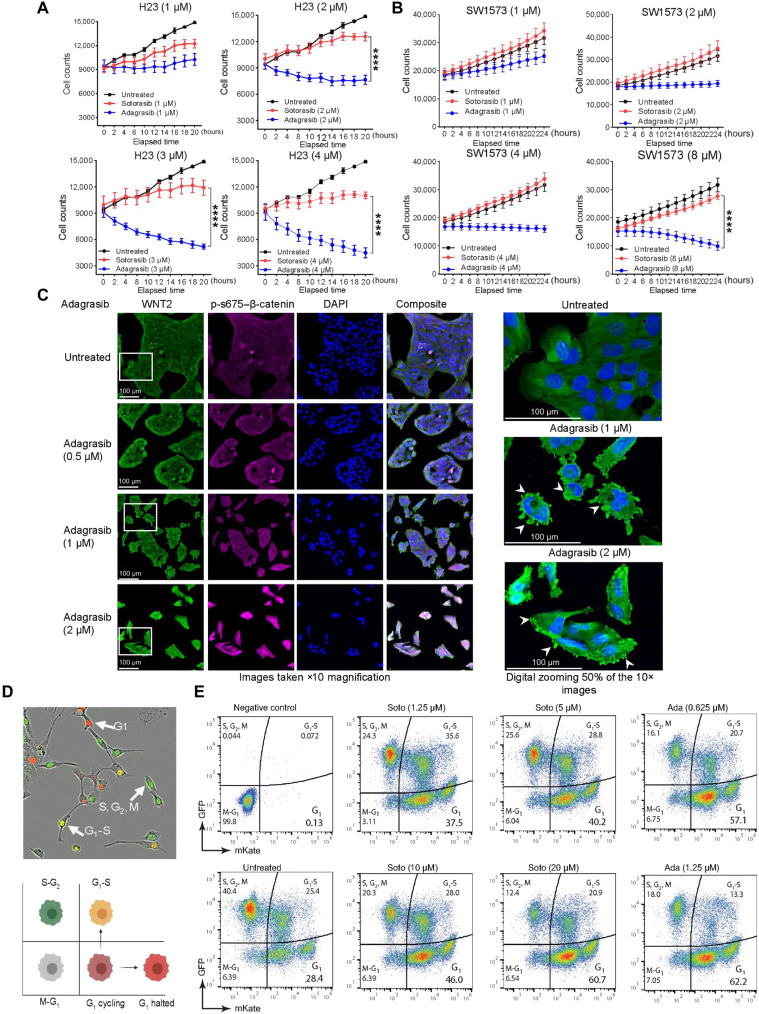
KRAS G12C inhibitors have a differential effect on cell growth and progression. (**A**) Effect of increasing concentrations (1 to 4 μM) of sotorasib and adagrasib exhibited different effects on cell proliferation over 24 hours in H23 cells. Two-way ANOVA was used for calculating statistical significance across various time points and for each drug concentration. *n* = 3. (**B**) Effect of KRAS inhibitors sotorasib or adagrasib increasing concentrations (1 to 8 μM) on SW1573 cells within 24 hours of drug treatment. Two-way ANOVA test was used to calculate the statistical significance. *n* = 3 sample per group. (**C**) Immunofluorescence image of SW1573 to support the dose dependent inhibitory effect of adagrasib on cell growth and expression of WNT2 (green) and phospsho-S675–β-catenin (magenta). The region of interest was zoomed 50% digitally to show membrane blebbing induced by adagrasib (white arrows). (**D** and **E**) Cell cycle dynamics were determined using IncuCyte Cell Cycle Lentivirus Reagent with fluorescence indicating cell cycle phase (brightfield image and schematic). Effect of increasing concentrations of sotorasib (1.25 to 20 μM) and adagrasib (0.6 to 1.25 μM) on cell cycle in SW1573 cells represented as pseudo color plots. The *y* axis of the plot represents events positive for GFP, and *x* axis represents the events positive for mKate2. mKate positive represents G_1_; GFP positive represents S, G_2_, and M; and double positive represents G_1_-S–transitioning cells.

Although both compounds are specific inhibitors of mutant KRAS G12C through covalent binding of the mutant cysteine residue, we observed that adagrasib retained activity against sotorasib-resistant cell lines, suggesting that the mechanisms underlying the inhibitory effect of the two drugs may be different. To explore the concentration and time needed for both the drugs to be effective, we treated the H23 and SW1573 cells with increasing concentrations of sotorasib or adagrasib and analyzed cell growth every hour for 24 hours in real time using IncuCyte (Fig. 5, A and B). At 4 μM sotorasib (red line graph, Fig. 5A) induced a cytostatic effect on the H23 cells, and the same concentration of adagrasib (blue line graph, Fig. 5A) induced a cytotoxic effect, as evident from the drop in the cell number. Similarly, in SW1573 cells, sotorasib treatment did not inhibit the cell growth even at a concentration of 8 μM, but under similar conditions, adagrasib induced cell death (Fig. 5B). Again, alluding to the fact that although both drugs are KRAS G12C inhibitors, they have different efficacies in KRAS G12C inhibition. To further validate the effect of adagrasib, we performed fluorescence staining of the SW1573 cells using the WNT2 and phospsho-S675–β-catenin antibodies. An equal number of cells were seeded, images were taken after 72 hours of the adagrasib treatment, and the differences in the cell number between the untreated and treated cells showed that a minimum of 1 μM adagrasib can efficiently block the proliferation of SW1573 cells. There was also an increase in the intensity of WNT2 (green) and phospsho-S675–β-catenin (Magenta) with increasing adagrasib concentration, which again suggests that the KRAS inhibition can increase the expression of WNT2 and activate CTNNB1 signaling. In addition, we observed that there was increased membrane blebbing in the SW1573 cells that were treated with 1 or 2 μM adagrasib. Membrane blebbing is usually correlated with apoptosis but could also arise due to loss of interaction with substratum (*33*). The significance of this blebbing in the SW1573 cells needs to be verified further using other apoptotic and focal adhesion markers (Fig. 5C).

### Adagrasib induces marked cell cycle arrest compared to sotorasib

In addition, we used flow cytometry to capture the differential effect of the two inhibitors on the cell cycle. We generated SW1573 stable cell lines expressing fluorescently tagged cell cycle markers using Essen Bioscience reagents (Materials and Methods). In the G_1_ phase, these cells expressed a red fluorescent protein, and in G_2_ or M, they expressed a green fluorescent protein. The cells transitioning from G_1_ to S phase expressed both proteins, resulting in yellow fluorescence (Fig. 5D, image). The Attune NxT Flow Cytometer and FlowJo V10 software were used to assess the cell distribution under untreated conditions. The gating strategy used to differentiate the cells in various stages of the cell cycle is mentioned in fig. S9 (A and B). Next, we treated the cells with increasing concentrations of sotorasib (1.25, 5, 10, or 20 μM) or adagrasib (0.6 and1.25 μM) for 72 hours and analyzed the changes in the cell cycle. We observed an increase in the percentage of the G_1_ population from ~28% (for untreated) to 60% following treatment with 20 μM sotorasib (Fig. 5E). Simultaneously, we observed a decrease in the S and G_2_-M populations, from 40% in untreated cells to 12% in cells treated with 20 μM sotorasib. However, in contrast to 20 μM sotorasib, adagrasib could induce an equivalent percentage of G_1_ accumulation at a minimum concentration of 0.6 μM, suggesting that adagrasib is an efficient inhibitor of the cell cycle (Fig. 5E).

**Fig. 6.**
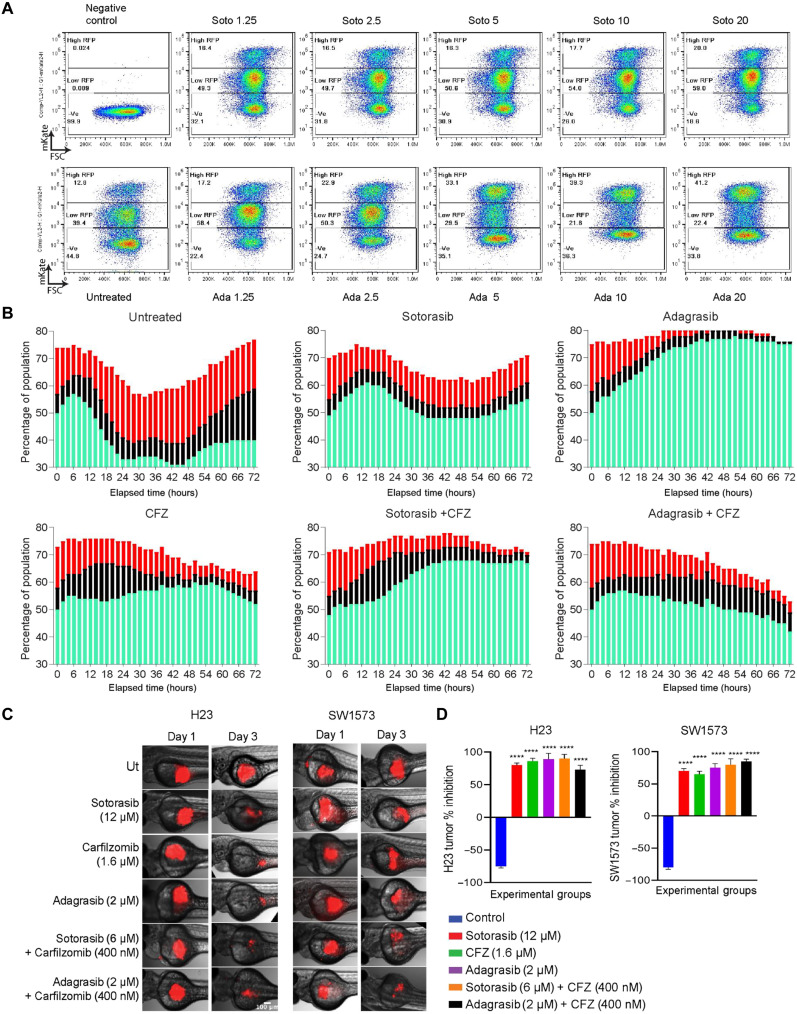
KRAS G12C inhibitors have a differential effect on the cell cycle. (**A**) Pseudo color plot representing the changes in the percentages of the G_1_ cell population with respect to drug treatment. The cells expressing low RFP (G_1_ cycling) and high RFP (G_1_ halted) are gated and analyzed for the increasing concentration of sotorasib (top row) and adagrasib (bottom row). The *y* axis of the plot represents events positive for mKate2, and *x* axis represents the forward scatter (FSC). (**B**) Cell cycle kinetics of the SW1573 cells was followed using the live cell imaging assay after drug treatment 10 μM sotorasib IC_50_ concentration, 10 μM adagrasib, 20 nM CFZ, sotorasib or adagrasib, and CFZ combination. Sotorasib and CFZ induced strong G_1_ arrest as done by adagrasib alone. The green bar represents G_1_ percent of the total population, the black bar represents S percent of the total population, and the red bar represents G_2_-M percent of the total population. (**C**) Red fluorescence dye–labeled H23 (left) and SW1573 (right) cells were xenotransplanted in zebrafish larvae, and images were taken after 3 days of 12 μM sotorasib, 2 μM adagrasib, 1.6 μM CFZ, 6 μM sotorasib + 0.4 μM CFZ, or 2 μM adagrasib + 0.4 μM CFZ treatment. (**D**) Percentage change in tumor growths was represented as bar graph against the experimental groups. The combination was effective at less concentration of the drugs. Statistical significance was calculated using one-way ANOVA. *n* = 10. ****P < 0.0001.

Furthermore, within the G_1_ population, we identified two distinct subgroups: one with low red fluorescent protein (RFP) expression (G_1_ cycling, hypothesized to participate in the cell cycle) and the other with high RFP expression (hypothesized to be arrested in the G_1_ stage) (Fig. 5D, cartoon). We analyzed the changes in these two subgroups with increasing concentrations of sotorasib and adagrasib. In untreated cells, the G_1_ population consisted of 39.3% low RFP and 12.8% high RFP, which transformed into 59% low RFP and 20% high RFP in the 20 μM sotorasib-treated cells. In contrast, 0.6 μM adagrasib increased the low RFP cell percentage to 58.4% and the high RFP population to 17.2%. Furthermore, upon increasing the concentration to 10 μM, the percentage of cells with low RFP decreased to 22.4%, and those with high RFP increased to 41.2% (Fig. 6A). These observations suggested that adagrasib could effectively push the cells more into G_1_ arrest state compared to sotorasib.

### Sotorasib and CFZ combination resembles adagrasib cell cycle kinetics

We used live cell imaging to investigate the effects of G12C inhibitors, either alone or in combination with CFZ, on cell cycle kinetics. The stable cell line generated for flow cytometry analysis was seeded on 96-well plates, and images were captured every 2 for 72 hours. In the untreated control cells at a time equal to 0 hours, 50% of the total population was in G_1_ phase (green bar), 7% contributed to the S phase (black bar), and 17% contributed to G_2_-M (red bar) phase. Between 0 and 72 hours, the changes in the different phases of the cell cycle are highlighted by the changes in the bar graphs, where the G_1_ oscillated between 31 and 57%, G_2_-S between 6 and 19%, and G_2_-M between 9 and 21%.

Upon treatment with sotorasib (10 μM), the G_1_ population fluctuated between 48 and 60%, while no significant changes were observed in the S or G_2_-M populations. In contrast, adagrasib treatment resulted in a 20% increase in the G_1_ population over the 72-hour period. The G_1_-S population decreased from 5 to 1%, and the G_2_-M population decreased from 7 to 0%, indicating a pronounced cell cycle arrest. Similarly, CFZ treatment led to a deceleration of the cell cycle, characterized by G_1_ accumulation and reductions in the S and G_2_-M populations. The combination of sotorasib and CFZ increased G_1_ population from 48 to 68% while simultaneously reducing the G_1_-S population to 3% and G_2_-M population to 1%, resembling the effect of adagrasib treatment (Fig. 6B). On the basis of these findings, we propose that adagrasib, in comparison to sotorasib, effectively inhibits the cell cycle and KRAS signaling. A similar effect was induced by a combining sotorasib and CFZ, which may explain the synergistic effect of both drugs.

### CFZ and sotorasib drug combination inhibited resistant tumor growth in zebrafish xenografts

Having confirmed the synergistic effect in 2D and 3D cell line models, we set out to determine the synergistic effects in vivo. We first used H23 and SW1573 cells to create zebrafish xenotransplants (see the Materials and Methods section) and compared changes in tumor size on days 0 and 3 by image analysis (Fig. 6C). For H23 cells, 12 μM sotorasib induced 75% of inhibition and CFZ induced 80% of inhibition, and a similar inhibition was induced by a combination of 6 μM sotorasib and 400 nM CFZ, again suggesting synergy. The same percentage of inhibition was induced by 2 μM adagrasib, and combining adagrasib with CFZ did not add much to the inhibition, suggesting that adagrasib as a single drug is more effective (Fig. 6D, first plot). For SW1573 cells, sotorasib, adagrasib, and CFZ were less effective at the same concentrations as in H23 cells. However, the combination of sotorasib or adagrasib with CFZ notably reduced the number of resistant tumors (Fig. 6D, second plot). Thus, sotorasib or adagrasib with CFZ acts synergistically when used in combination, as the KRAS inhibitor dose can be reduced two- to fourfold. However, compared with sotorasib, adagrasib alone was more effective in terms of tumor reduction.

### CFZ and sotorasib drug combination inhibited resistant tumor growth in vivo

**Fig. 7.**
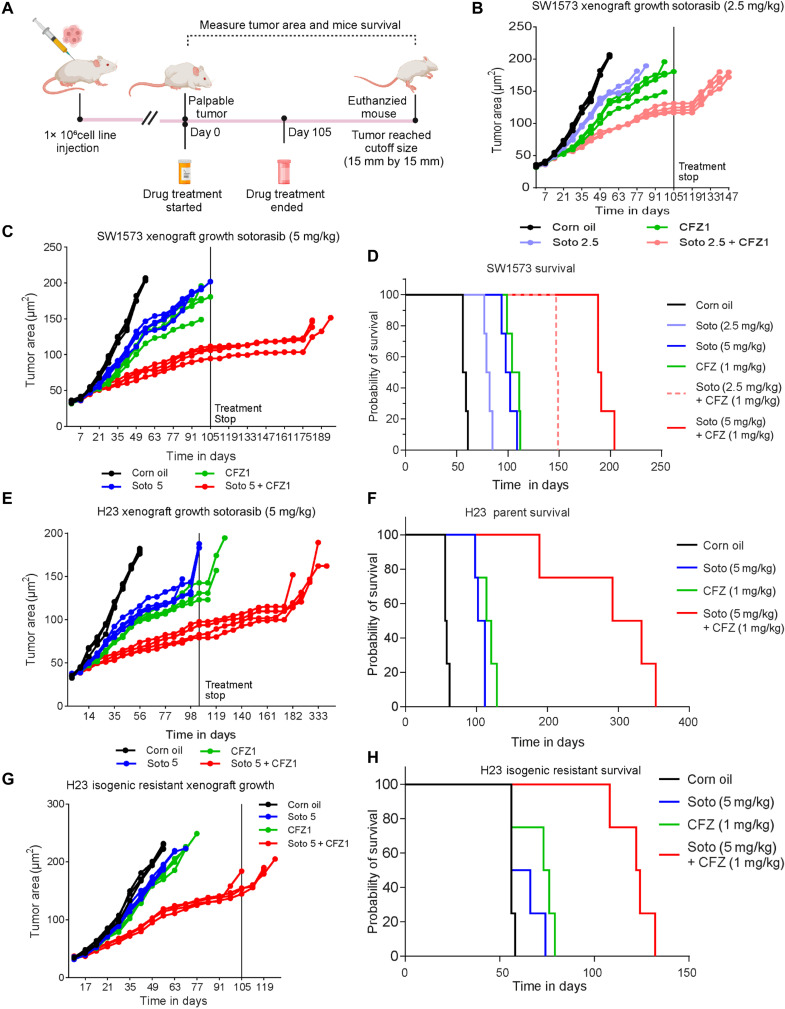
In vivo models confirm the sensitivity of cells to KRAS G12C inhibitors and CFZ combination treatment. Mice xenografts were created using SW1573 cells to determine the antitumor effects of sotorasib, adagrasib, and CFZ and their combinations in vivo. (**A**) Schematic of in vivo study. (**B** and **C**) Changes in the tumor area changes (mm^2^) of xenografts with respect to sotorasib (2.5 or 5 mg/kg) or CFZ (1 mg/kg) single treatments and drug combination treatments [sotorasib (2.5 mg/kg) + CFZ (1 or 5 mg/kg) + CFZ (1 mg/kg)]. Statistical significance was calculated using one-way ANOVA. *n* = 5. ****P < 0.0001. (**D**) Survival probability of the mice harboring SW1573 cell line–derived xenografts. The combination treatment of sotorasib (2.5 mg/kg) + CFZ (1 or 5 mg/kg) + CFZ (1 mg/kg) has the highest median survival compared to single treatments. (**E** and **F**) Changes in the tumor area (mm^2^) of H23 cell line–derived xenografts with respect to sotorasib (5 mg/kg) or CFZ (1 mg/kg) single treatments or combination treatments [sotorasib (5 mg/kg) + CFZ (1 mg/kg)]. Survival probability of the mice harboring H23 cell line–derived xenografts. Statistical significance was calculated using one-way ANOVA. *n* = 5. ****P < 0.0001. (**G** and **H**). Changes in the tumor area (mm^2^) of H23 isogenic resistant cell line–derived xenografts and survival. The combination treatment of sotorasib (5 mg/kg) + CFZ (1 mg/kg) has the highest median survival compared to the single drug. Statistical significance was calculated using one-way ANOVA. *n* = 5. ****P < 0.0001.

The effects of sotorasib, CFZ, and their combinations on survival were evaluated by creating mouse xenografts using the inherently resistant SW1573 cells. One million cells were injected into the animals, and after the palpable tumor was observed, the mice were randomly divided into six groups, each consisting of five mice. The animals were treated with corn oil, sotorasib (2.5 mg or 5 mg/kg body weight), CFZ (1 mg/kg body weight), sotorasib (2.5 mg) + CFZ (1 mg/kg body weight), or sotorasib (5 mg) + CFZ (1 mg/kg body weight). The treatment was administered for 105 days, and after treatment, the animals were monitored for tumor growth until it reached a maximum size of 15 mm by 15 mm. Survival was calculated from day 0 of treatment until the mouse was euthanized, as shown in the schematic (Fig. 7A). The average weight gain among the six groups of mice was similar, indicating no gross toxic effects of the drugs.

The changes in tumor area were measured from the initiation of drug treatment until the mice were euthanized. Tumor growth was inhibited by sotorasib, where the 5 mg/kg body weight (b.w.) dose of sotorasib effectively delayed tumor growth compared to the 2.5 mg/kg b.w. dose of sotorasib (Fig. 7, B and C). Similar inhibition of tumor growth was observed in the CFZ-treated group, where CFZ effectively reduced the growth rate of xenografts compared to the untreated group (green line graph, Fig. 7, B, and C). However, the combination of sotorasib and CFZ showed a highly significant inhibition of tumor growth compared to either drug used alone (red line graph, Fig. 7, B and C).

Likewise, the median survival age of the mice was calculated where the mice treated with corn oil was the shortest, i.e., 56 days, followed by those treated with sotorasib alone at doses of 2.5 mg/kg b.w. (80 days) or 5 mg/kg b.w. (100 days). The CFZ treatment alone also exhibited an equivalent survival effect of (107 days) compared to sotorasib. In both drug combination studies, i.e., sotorasib (2.5 mg/kg b.w.) with CFZ (1 mg/kg) or sotorasib (5 mg/kg b.w.) with CFZ (1 mg/kg b.w.), the survival rate was the highest. The median survival age for sotorasib (2.5 mg/kg b.w.) with CFZ (1 mg/kg b.w.) was 148 days, and for sotorasib (5 mg/kg b.w.) with CFZ (1 mg/kg b.w.), it was 189 days (Fig. 7D). The SW1573 xenografts treated with sotorasib (5 mg/kg) and CFZ (1 mg/kg) survived for an additional 93 days compared to 4 days for sotorasib alone or 11 days for CFZ alone after the end of the treatment. These data suggested a synergistic effect of CFZ + sotorasib, which recapitulated the in vitro findings. CFZ and sotorasib drug combination synergistically inhibited tolerant tumor growth in vivo. The effect of adagrasib or CFZ alone or in combination was also analyzed in in vivo experiments and showed an additive effect (fig. S10, A to D).

### CFZ and sotorasib drug combination inhibited tolerant tumor growth in vivo

Next, an in vivo experiment was conducted using H23 parental or isogenic resistant cells resistant to sotorasib. In this study, we divided the animals into four groups and administered corn oil, sotorasib (5 mg/kg b.w.), CFZ (1 mg/kg b.w.), or a combination of both sotorasib (5 mg/kg b.w.) and CFZ (1 mg/kg b.w.). We measured changes in tumor area and observed a statistically significant reduction in H23-derived xenografts with sotorasib or CFZ treatment alone, but the combination exerted the strongest inhibition (red line graph, Fig. 7E). The median survival duration after treatment with corn oil alone was 56 days, whereas it was 107 days with sotorasib alone, 117 days with CFZ alone, and 312 days with the combination (Fig. 7F). Thus, the mice treated with the combination survived an additional 216 days compared to 11 days for sotorasib or 21 days for CFZ alone, indicating a synergistic effect of the drug combination on tumor suppression and mouse survival. However, sotorasib or CFZ could not inhibit tumor growth in xenografts induced by isogenic resistant cells, but the combination remarkably reduced tumor growth (red line graph, Fig. 7G). In isogenic resistant xenografts, the median survival for the corn oil–treated group was 56 days, 61 days for the sotorasib-only group, and 74 days for CFZ alone group, confirming the ineffectiveness of the drugs when used individually. The mice could not reach the end of the treatment period when sotorasib or CFZ was given alone. However, the median survival of mice treated with the drug combination was 123 days, representing a better survival outcome compared to the single drugs (Fig. 7H). Thus, all three studies concluded that the combination of sotorasib and CFZ exhibited a synergistic effect in reducing tumor burden and increasing survival rates.

## DISCUSSION

Sotorasib and adagrasib are highly promising mutant selective KRAS G12C inhibitors. However, resistance to these inhibitors, whether innate or acquired, has already emerged as a serious concern (*14*–*20*, *34*, *35*), emphasizing the importance of an unbiased and in-depth understanding of the resistance mechanism(s). Resistance is generally thought to be maintained primarily through random genetic mutations and the subsequent expansion of mutant clones via Darwinian selection (*36*, *37*). Hence, this phenomenon has historically been approached from a reductionist and gene-centric perspective (*38*). However, it is now evident that therapy resistance can also arise from heterogeneous drug-tolerant persister cells or minimal residual disease through both genetic and nongenetic mechanisms (*39*, *40*). To further characterize the innate and acquired resistance, we used three different KRAS-G12C NSCLC cell lines, namely, H358, H23, and SW1573, that had different genetic backgrounds and responses to sotorasib. All three cell lines were confirmed to have KRASG12C and p53 mutations, and in addition, H23 cells have STK11 and ATM mutations, whereas SW1573 cells have CDKN2A, SMAD4, CTNNB1, PIK3CA, and SMARCB1 mutations (Cellosaurus database CVCL_1547, CVCL_1720, and CVCL_1559). Very often, STK11 is a frequent co-occurring mutation detected in patients with KRAS mutant, followed by ATM mutations, and the survival chances of patients having these mutations were reported to be very poor (*41*, *42*). In contrast, the PIK3CA and CTNNB1 mutations found in SW1573 occur less frequently in KRAS-mutant NSCLC (*41*).

**Fig. 8. F8:**
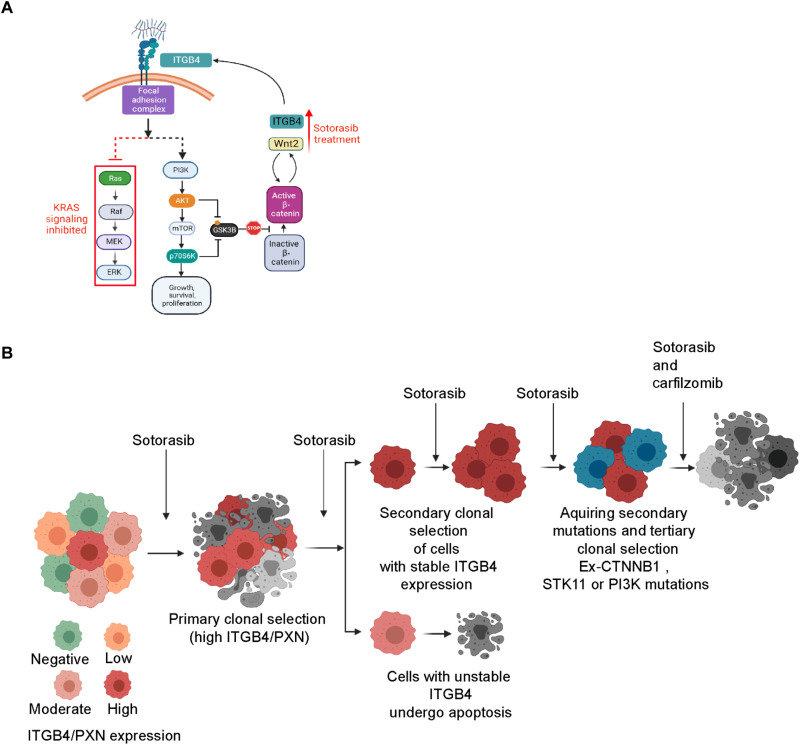
Schematic representation of the signaling changes and evaluation of acquired resistance. (**A** and **B**) A cartoon representation summarizing the cellular signaling involvement in sotorasib and the evolution of acquired resistant clones under continuous pressure. The cartoons were made using BioRender. PI3K, phosphatidylinositol 3-kinase. MEK, MAPK kinase.

The H358 cells having two mutations (KRASG12C and p53) were observed to be very sensitive to sotorasib, whereas H23 cells having four mutations were tolerant, and SW1573 cells having five mutations were inherently resistant. Tolerant cells often have a heterogeneous mixture of sensitive and persister cells, and under continuous selection pressure, these persister cells contribute to acquired resistance. Consistent with this hypothesis, we were able to generate isogenic resistant H23 cells that could tolerate to sotorasib up to 20 μM without having any gain in function mutations in genes reported by Awad *et al.* (*14*) or the mutations observed in the SW1573 cells. However, these resistant cells showed changes in expression, interactions, and signaling associated with ITGB4 and β-catenin. The increased ITGB4 expression supported AKT activation, which in turn suppresses GSK-3β function and activates β-catenin, and this acts as a bypass signaling mechanism to overcome sotorasib toxicity (Fig. 8A). Thus, the transition from tolerant to overtly resistant could be driven by nongenetic mechanisms. There is a likelihood that patients having KRAS G12C and STK11 comutations may respond to sotorasib initially but eventually will develop resistance and be nonresponsive to sotorasib (*39*–*43*). However, our results also indicated that patients having a mutation in the genes contributing to bypass pathways such as PIK3CA and CTNNB1 may be inherently resistant to sotorasib similar to the SW1573 cells. Overall, the present study suggests that all tumors may not evolve to a resistant state similar to H358 cells. Some may evolve from a sensitive to a tolerant state through nongenetic mechanisms, and some may evolve to a highly resistant state similar to SW1573 cells by acquiring mutations in the stressed network (Fig. 8B) (*39*, *44*). Thus, scenarios underscore the genetic/nongenetic duality of drug resistance in cancer (*39*).

The acquired resistance to sotorasib can be reversed by using the combination of CFZ and sotorasib (Fig. 8B). This combination abrogated the expression of ITGB4 and β-catenin and their downstream signaling, leading to increased sensitivity[Fig F8]. Our motivation to discern the efficacy of CFZ in alleviating sotorasib resistance was based on our previous finding where it was effective in alleviating cisplatin resistance in a mutant KRAS LAUD cell line (*26*). Although found as a proteasome inhibitor, we suspect that CFZ perturbs expression and signaling downstream of ITGB4/PXN and WNT/β-catenin, which is independent of proteasomal inhibition. Thus, it can be surmised that the nuanced role of focal adhesion complex components and WNT/β-catenin signaling can be suppressed by the sotorasib and CFZ combination.

The protein interaction networks play a critical role in the nongenetic mechanism of resistance by virtue of their ability to “rewire” the network (*44*, *45*). The conformational dynamics of proteins, especially of IDPs that occupy hub positions in these cellular circuitries, affect several cellular signaling pathways (*46*). Although KRAS may not be a bona fide IDP, it can be considered as a “hybrid” protein. Specifically, the G domain (residues 1 to 166), which is regarded as the functional domain, is mostly ordered. However, there are several short, disordered regions interdigitated between ordered regions, such as the P loop, switch I, and switch II regions, which contribute to conformational preferences. Thus, the apo form of the KRAS molecule exists as a conformational ensemble with considerable flexibility, owing to its disordered regions (*30*, *32*). By contrast, the ensemble was significantly structured upon GDP/guanosine 5′-triphosphate binding. Notably, various point mutations in KRAS bias the conformational preferences of the holoenzymes. Therefore, although functionally mutant-selective inhibitors impair GTPase activity (lock it in the unbound state) and render the oncoprotein inactive, they affect its malleability, thereby affecting downstream signaling with potentially different clinical outcomes. Notably, the most common mutations (codons 12, 13, and 61) occurred in the P loop (residues 10 to 14) and switch II regions (residues 58 to 72), respectively, further underscoring the crucial role of the conformational changes and downstream signaling. Consistent with this argument, the adagrasib-bound KRAS G12C may have a different conformation compared to the sotorasib-bound molecule, which could potentially impinge on the partners with which they interact differently. This may account for the differences in their efficacy and mechanisms of action. Notably, we consistently observed differences in the molecular weights of the drug-free KRAS molecule, sotorasib-bound KRAS, and adagrasib-bound KRAS using SDS gel electrophoresis. This is likely due to conformational differences and/or posttranslational modifications, such as phosphorylation, because the two small-molecule inhibitors differ only by ~200 Da, yet the observed differences are 1 to 2 kDa (Fig. 1E).

In summary, we have shown that cells activate ITGB4 and β-catenin signaling as the bypass mechanism for acquiring resistance against sotorasib. Nonetheless, the resistance developed against sotorasib may not be effective against adagrasib. Together, these results highlight the role of nongenetic mechanisms of drug resistance in cancer and call for closer attention to be paid to the differences in interactions of the various drug-bound ensembles of mutant KRAS. Overall, our findings unveil previously unrecognized nongenetic mechanisms underlying resistance to sotorasib and propose a promising treatment strategy to overcome resistance.

## MATERIALS AND METHODS

### Cell lines and reagents

NSCLC cell lines (H23, H358, and SW1573) were obtained from American Type Culture Collection (Manassas, VA, USA). NSCLC cell lines were cultured in RPMI 1640 medium (Corning) supplemented with fetal bovine serum (10%), l-glutamine (2 mM), penicillin/streptomycin (50 U/ml), sodium pyruvate (1 mM), and sodium bicarbonate (0.075%) at 37°C and 5% CO_2_. CFZ and sotorasib (AMG 510) were purchased from Selleck Chemicals (Houston, TX, USA). MRTX849 was purchased from MedChemExpress (Monmouth Junction, NJ, USA).

### Antibodies

Antibodies against ITGB4 (catalog no. 4707), FAK (catalog no. 3285), γH2AX (catalog no. 2577), p27 (catalog no. 3686), phospho-Rb (S807/811) (catalog no. 8516), phospho-AKT (S473) (catalog no. 4060), AKT (catalog no. 4685), phospho-Src (Y416) (catalog no. 6943), Src (catalog no. 2109), phospho-ERK (T202/Y204) (catalog no. 4377), ERK (catalog no. 4695), phospho–β-catenin (S675) (catalog no. 9567), β-catenin (catalog no. 8480), phospho-p70 S6K (T389) (catalog no. 9234), p70 S6K (catalog no. 2708), phospho–GSK-3β (S9) (catalog no. 9336), cleaved PARP(Asp^214^) (catalog no. 5625), SOS1 (catalog no. 5890), AXIN1 (catalog no. 2087,C76H11), and MYCtag (catalog no. 2276) were purchased from Cell Signaling Technology (Danvers, MA, USA). KRAS antibody (catalog no. LS-C175665100) was purchased from LifeSpan BioSciences (Seattle, WA, USA). PXN antibody (catalog no. AHO0492) was purchased from Invitrogen (Waltham, MA, USA). WNT2 antibody (catalog no. 66656-1-Ig) was purchased from Proteintech (Rosemont, IL, USA). β-Actin antibody was purchased from Sigma-Aldrich (catalog no. A5441) (St. Louis, MO, USA). The following antibodies were also used: CCND1 (catalog no. MA5-14512, Invitrogen, Rockford, IL, USA), CDK2 (catalog no. 10122-1-AP, Proteintech, Rosemont, IL, USA), BRAF (Serine /Threonine protein kinase B-Raf) (catalog no. SC-5284, F-7, Santa Cruz Biotechnology Inc.), and glyceraldehyde-3-phosphate dehydrogenase (GAPDH) (catalog no. SC-47724, clone 0411, Santa Cruz Biotechnology Inc., Dallas, TX, USA Dallas, TX, USA).

### Immunoblotting

Cell lysates were prepared with 1× radioimmunoprecipitation assay buffer (MilliporeSigma) and denatured in 1× reducing sample buffer at 95°C for 5 min. Protein samples (15 μg) were run on 4 to 15% Criterion TGX gels (Bio-Rad, Hercules, CA, USA) and transferred onto nitrocellulose membranes (Bio-Rad). Blots were blocked with 5% nonfat milk in TBS-T (Tris buffered saline with Tween 20) for 1 hour at room temperature and probed with primary antibody diluted in 2.5% bovine serum albumin in TBS-T overnight at 4°C. After three washes with TBS-T, blots were incubated with horseradish peroxidase (HRP)–conjugated secondary antibodies for 2 hours at room temperature. After three more washes, bands of interest were visualized via chemiluminescence using Western Bright ECL HRP substrate (Advansta, Menlo Park, CA, USA) and imaged with the ChemiDoc MP imager (Bio-Rad). The gels were run in parallel to detect multiple antigens to gather comprehensive information on the signaling changes. All the individual blots were arranged in Powerpoint and were processed to reduce 5% brightness, and the complete set of blots was copied to Illustrator to prepare the figures.

### Quantitative real-time PCR and RNA-seq

Real-time qPCR reactions were performed using TaqMan Universal PCR Master Mix (Thermo Fisher Scientific, Waltham, MA) and analyzed by the Quant Studio7 Real-time PCR system (Life Technologies, Grand Island, NY). Total RNA isolation and on-column deoxyribonuclease digestion from cells were performed on the basis of the manufacturer’s protocol RNeasy Plus Mini Kit (catalog no. 74134, QIAGEN). A total of 1 μg of RNA was used to synthesize the cDNA according to the one-step cDNA synthesis kit from QuantaBio (catalog no. 101414-106). TaqMan probes for 18*S* (Hs03003631_g1), WNT2 (HS00608222_m1), CFTR (HS00357011_m1), and CCL2 (HS00234140_m1) were purchased from Thermo Fisher Scientific (Waltham, MA). The mRNA expression was analyzed using multiplex PCR for the gene of interest and 18*S* ribosomal RNA as reference using two independent detection dyes, FAM (Fluorescein amidites) probes and VIC (2'-cholor-7'-phenyl-1,4-dichloro-6-carboxyfluorescein) probes, respectively. Relative mRNA expression was normalized to GAPDH signals and calculated using the Δ Ct method. The gene expression of the rest of the 17 genes was done using the SYBR qPCR methods. The SYBR Green primers for the 17 genes that were designed and ordered from Integrated DNA technologies are as follows: MYOCD (forward: GCAACACCGATTCAGCTACCTAG; reverse: GGTATTGCTCAGTGGCGTTGAAG), PRXX1 (forward: TGCAGGCTTTGGAGCGTGTCTT; reverse: CTCATTCCTGCGGAACTTGGCT), BAMBI (forward: TACAGAGGGCTGCACGATGTTC; reverse: AAGTCAGCTCCTGCACCTTGGT). TNFRSF (forward: GGTGCATTCTGCAGCCAGTCTT; reverse: CAGGCATCTGAAAACTCGCCAC), MEOX1 (forward: GAGATTGCGGTAAACCTGGACC; reverse: TCTGAACTTGGAGAGGCTGTGG), STC (forward: GCAGGAAGAGTGCTACAGCAAG; reverse: CATTCCAGCAGGCTTCGGACAA), CTTNBP2 (forward: TAACCACGCCAACAGAGAAGGC; reverse: GCACTTGTCTCTCCTTTCTGGC), DCLK1 (forward: ACCGATGCCATCAAGCTGGACT; reverse: TCCTGGTAACGGAACTTCTCCG), Y123 (forward: CAACCTCTACGCTGCCGAGTCG; reverse: AAAGTCTCCTTGCAGGAG), CRH (forward: AGAGAAAGGCGGTCCGAGGAG; reverse: GTGAGCTTGCTGTGCTAACTGC), SHH (forward: CCGAGCGATTTAAGGAACTCACC; reverse: AGCGTTCAACTTGTCCTTACACC), DUSP4 (forward: TACTCGGCGGTCATCGTCTACG; reverse: CGGAGGAAAACCTCTCATAGCC), COL26 (forward: AAGAAGGAGAGAAAGCCGCCAC; reverse: ATGTGCTCCAGGATGAGGACTC), NTSR1 (forward: GTCATCGCCTTTGTGGTCTGCT; reverse: GAAGAGTGCGTTGGTCACCATG), UBEQ (forward: TTAGCGACCGCTTCATCTCCGT; reverse: GGATGAACTCGGTGTTGGTCTC), RSPO3 (forward: CCAGAAGGGTTGGAAGCCAACA; reverse: CCTTCTTCGTGCATGGACTCCA), NT5E (forward: AGTCCACTGGAGAGTTCCTGCA; reverse: TGAGAGGGTCATAACTGGGCAC), and SERPINB2 (forward: GCTGTTTGGTGAGAAGTCTGCG; reverse: RCTGCACATTCTAGGAAGTCTACT).

RNA was extracted from both H23 cells untreated (parent), H23 sotorasib treated (parent treated), and H23 cells resistant to 7.5 μM treated with sotorasib (resistant treated). The RNA was quantified and sent to Integrative Genomics Core at the City of Hope for RNA-seq. Reads were aligned against the human genome (hg38) using STAR [version 2.5, (*47*)]. Read counts were quantified using htseq-count [version 0.9.1, (*48*)], with UCSC Known Gene annotations [TxDb.Hsapiens.UCSC.hg19.knownGene, downloaded 30 August 2018 (*49*)]. Fold change values were calculated from fragments per kilobase per million reads (FPKM) (*50*)–normalized expression values, which were also used for visualization (following a log_2_ transformation). Aligned reads were counted using GenomicRanges (*51*). RSeQC (version 2.6.6) (*52*) showed no substantial bias in the coverage of RNA-seq reads. For example, transcript integrity number (TIN) (*53*) scores are above 70 for all samples, with TIN scores annotated in the Gene Expression Omnibus (GEO) deposit (GSE 192619). A total of 40 million reads were analyzed for each condition. *P* values were calculated from raw counts using edgeR (version 3.16.5) (*54*), and false discovery rate (FDR) values were calculated using the method of Benjamini and Hochberg (*55*). Before *P* value calculation, genes were filtered to only include transcripts with an FPKM expression level of 0.1 (after a rounded log_2_ transformation) in at least 50% of samples (*56*) and genes that are greater than 150 base pairs. Genes were defined as differentially expressed if they had a |fold change| > 1.5 and FDR < 0.05. While the code has to be modified for every project (with multiple rounds of analysis and discussion before deciding upon a final set of results), these scripts are a modified version of a template for RNA-seq gene expression analysis (https://github.com/cwarden45/RNAseq_templates/tree/master/TopHat_Workflow).

Overlap between the comparisons for parent and resistant, each separately compared to the control samples. GSEA (v2.2.2) (*57*) was used to calculate enrichment of MSigDB Hallmark genes sets (version 7.0) (*58*), with -rnd_seed 0 and -permute gene_set (due to only having duplicates). In addition, Enrichr tables for MSigDB Hallmark gene sets (described under “Pathways” as “MSigDB Hallmark 2020”) were downloaded from the web interface (on 3 September 2021), and a custom R script was used to plot signed −log_10_(FDR) values (positive for up-regulated genes and negative for down-regulated genes). Volcano plots for RNA-seq data were created using a custom R script. Heatmaps were created using heatmap.2 from the “gplots” package (version 3.1.0, Pearson dissimilarity was used as the distance metric).

### Genomic sequencing and analysis

Genomic DNA was extracted using the QIAGEN Genomic DNA Isolation Kit from the parental H23 cells and from the H23 cells resistant to 7.5 or 20 μM sotorasib. The quality check on the DNA was performed by the genomic core of the City of Hope, and then exome sequencing was done for a total of 40 million reads. The cutadapt v1.18 was used to remove all unwanted sequences including adapter, primer, and polyadenylate tails from the whole-exome sequencing reads. The processed reads were aligned to the human reference genome (GRCh38) using Burrows-Wheeler Aligner v0.7.17 (https://github.com/lh3/bwa), and PCR duplicates were removed by Mark Duplicates algorithm in Picard (v2.21; https://broadinstitute.github.io/picard/). We then performed base quality score recalibration and local realignments around known insertion deletion (INDEL) sites with GATK v4.1.8.0 (*59*). We used MuTect2 in GATK to identify somatic single-nucleotide variants and INDELs (*60*). We also performed “LearnReadOrientationModel”, “GetPileupSummaries” and “Calculate Contamination” in GATK to find orientation bias and to estimate cross-sample contamination and removed biased and contaminated variants using FilterMutectCalls in GATK. For better detection of somatic variants, we also performed samtools mpileup v1.10 (*61*) and varcan v2.4.4 (*62*) with union list of detected somatic variants from all tumor samples. table_annova.pl in ANNOVAR (*54*) was used to annotate confident variants, and maftools R package v2.7.41 (https://genome.cshlp.org/content/28/11/1747) was used to summarize and visualize annotated somatic variants.

### Cell proliferation and apoptosis assay

Cell proliferation assays were performed using cell lines stably transfected with NucLight Red Lentivirus (Essen BioScience) to accurately visualize and count the nucleus of a single cell. Cells were seeded on a 96-well plate and allowed to adhere for 24 hours. Test compounds were added at indicated concentrations. Caspase 3/7 Green Apoptosis Reagent (Essen BioScience) was also added as a green fluorescent indicator of caspase 3/7–mediated apoptotic activity. To monitor cell proliferation and apoptosis over time, the plate was placed in the IncuCyte S3 Live Cell Imaging System (Essen BioScience) and images were acquired every 2 hours. Data analysis was generated by the IncuCyte software using a red fluorescence mask to accurately count each cell nucleus and a green fluorescence mask to measure apoptosis over time. 3D spheroid assay experiments were performed using cell lines stably transfected with NucLight Red Lentivirus (Essen BioScience) to visualize red fluorescence as an indicator of cell viability. Cells were seeded on a 96-well ultralow attachment plate and allowed to form spheroids overnight. Drug treatment was added as indicated along with caspase 3/7 green apoptosis reagent (Essen BioScience), used as a green fluorescence indicator of cell death due to loss of cell membrane integrity. To monitor cell proliferation and apoptosis over time, the plate was placed in the IncuCyte, and images were acquired every 2 hours. Data analysis was generated by the IncuCyte software using a red fluorescence mask to accurately measure intensity and area of red fluorescence, indicating spheroid viability, and a green fluorescence mask, indicating cell death.

### Knockdown and proliferation assays

The KRAS G12C–mutated cells were seeded on each well of a six-well plate and transiently transfected with a control (scrambled) or gene-specific siRNA once the cells reach 80% confluency. Twelve hours after the transfection, the medium was removed and the cells were harvested, counted, and seeded in a 96-well plate. The proliferation of these cells on a 96-well plate was determined using the IncuCyte S3 Live Cell Imaging System (Essen Bioscience, Ann Arbor, MI). Knockdown of Scramble (#SR30004), ITGB4 (#SR302473CL), PXN (SC-29439, Santa Cruz Biotechnology Inc., TX, USA), and CTNNB1 (#SR319832) at the mRNA level was executed using siRNAs purchased from OriGene (Rockville, MD, USA). JetPRIME transfection reagent (Polyplus-transfection, Illkirch, France) was used to transfect the siRNAs according to the manufacturer’s protocol. To generate the WNT2 KO cell lines, we used the WNT2 CRISPR-Cas9 KO plasmid (#SC-402665) and WNT HDR plasmid (#SC-402665-HDR) from Santa Cruz Biotechnologies Inc). The H23 or SW1573 cells were transfected with plasmids, and stable cell line selection was done using puromycin. The cells were sensitive to the KO, and isolating a single KO clone was difficult. The mixture of transient KO cells was used for the proliferation and sotorasib inhibitory assay.

### ITGB4 overexpression and stable cell line generation

The lentiviral particle for expressing ITGB4 under the cytomegalovirus promoter was purchased from Genecopia (LPP-Z3028-Lv128-100). The backbone of the vector was pReceiver-Lv128, and the selection marker was puromycin. The H23 cells were seeded at a density of 20,000 cells per well of a 96-well plate, and once the cells adhere, 10 μl of the lentiviral particle was used for infecting the cells in the presence of polybrene (4 μg/ml; sc-134220, Santa Cruz Biotechnologies Inc). After 48 hours of infection, the medium was replaced with fresh medium, and the cells were allowed to grow. The cells were expanded to an approximate density of 1 million cells/ml, and drug selection was done using puromycin dihydrochloride (1 μg/ml; A113803, Thermo Fisher Scientific, USA). The selected cells were tested for the expression of ITGB4 expression, and once the expression was confirmed, they were used for the experimental purpose.

### Combination index

For combination index (CI) calculation, incured cell lines were seeded in 96-well plates with 5000 cells per well. Three biological replicates (three 96-well plates for each drug combination) were used. For both cell lines, two drug combinations were used, sotorasib and CFZ. The drugs were used in linear dilution series with a dilution factor of 2. Sotorasib doses ranged from 0 to 64 μM and CFZ from 9.5 to 608 nM. The plates were read at 72 hours using the IncuCyte Live Cell Analysis System to measure live cells (incurred object count per well). We then use an R package called SynergyFinder (*63*, *64*) to find the nature of drug-drug interaction (i.e., if they work in synergy or antagonistically or noninteractively). For this purpose, the drug response matrix is supplied to the mentioned package, which then uses several models, namely, highest single agent (*65*), Loewe additivity (*66*), Bliss independence (*67*), and zero interaction potency (*68*) to quantify the degree of drug synergy. The dose response matrix was used to calculate individual CI.

### Statistical analysis

The experiments were reputed a minimum of three times to generate a conclusion. One-way analysis of variance (ANOVA), two-way ANOVA or nonlinear regression, or simple was performed to calculate the significance between datasets as indicated within each result or figure legend. A level of significance of *P* < 0.05 was chosen. Data are presented as means with SD (± SD) in all figures in which error bars are shown. Graphs were generated using GraphPad Prism 7 software.

### Zebrafish xenotransplant experiments

All zebrafish experiments were carried out in accordance with a protocol approved by the Institutional Animal Care and Use Committee [(IACUC) #18119]. NSCLC cell line H23 (KRAS G12C heterozygous) was seeded in a six-well plate until 60 to 70% confluency. One day before microinjection, H23 or SW1573 cells were stained with DiI (fluorescent lipophilic cationic indocarbocyanine) green dye. On the day of microinjection, the 48–hours postfertilization (hpf) zebrafish larvae were dechorionated to release the larvae. The larvae were anesthetized using tricaine (MS-222) at a final concentration of 200 μg/ml (stock, 10 mg/ml). The larvae were left in anesthetic for 1 to 2 hours until they were motionless for efficient microinjection. The cells were trypsinized, and cell numbers were counted using a cell counter (Nexcelom Bioscience Cellometer Auto T4). The cells were made into a homogenous suspension with 10 cells/nl. The cells were injected in the perivitelline space of anesthetized 48-hpf larvae (180 to 200 cells/nl) using Nanoject III manual programmable nanoliter injector. The 24-hpi zebrafish xenografts were screened for the formation of an obvious bolus of cancer cells (tumor) using a fluorescence microscope. The larvae were distributed in a 96-well plate with different treatment sets (untreated and drug treated: sotorasib, adagrasib, and CFZ single treatment and combination treatment with CFZ). Drug toxicity effects on growth and development were also assessed by examining the length and shape of the zebrafish body. For the untreated sample set, the larvae were left in embryo medium throughout the experiment. The larvae were imaged using Zeiss Observer 7 microscope (5× objective) for days 1 and 3 of microinjection. The images were processed using Fiji imaging software. The percent inhibition of the tumor growth was calculated. The fluorescent intensity was measured using Fiji image software by thresholding the green channel using Otsu’s setting. The intensity of days 1 and 3 was compared to calculate the inhibition percentage. The error bars represent (±SD). *n* = 10 untreated larvae and *n* = 15 treated larvae were used for each condition.

### Mouse xenograft studies

Athymic nude nu/nu mice were obtained from Charles River Laboratories (Wilmington, MA) and were acclimated for a week before the experiment. All animal experiments were carried out in accordance with a protocol approved by the IACUC (#16004). Thirty 8-weeks-old mice were divided into six groups of five animals [treatment with: (i) corn oil i.e., vehicle control; (ii) sotorasib (2.5 mg/kg b.w.), (iii) sotorasib (5 mg/kg b.w.), (iv) CFZ (1 mg/kg b.w.); (v) sotorasib (2.5 mg/kg b.w.) + CFZ (1 mg/kg b.w.); and (vi) sotorasib (5 mg/kg b.w.) + CFZ (1 mg/kg b.w.)]. All 30 animals were injected with 1 × 10^6^ SW1573 cell suspensions in 100 μl of phosphate-buffered saline subcutaneously into one flank of each mouse. Animals were randomized into treatment groups. Treatment was started 12 days after the SW1573 NSCLC cells implantation to discern palpable tumor growth. Treatments were given to mice via oral gavage twice a week for 16 weeks. Animals were examined daily for signs of tumor growth. Tumors were measured in two dimensions using calipers, and body weights were recorded weekly after treatment. The mice were euthanized by CO_2_ asphyxiation followed by cervical dislocation once the tumor reaches the maximum size of ~200 mm^2^. A second study for the combination effect of sotorasib and CFZ was done using the xenografts obtained from H23 parental and H23 isogenic resistant lines. Twenty 8-week-old mice harboring H23 cell line derived xenografts were divided into four groups of five animals [treatment with: (i) corn oil, i.e., vehicle control; (ii) sotorasib (5 mg/kg b.w.); (iii) CFZ (1 mg/kg b.w.), and (iv) sotorasib (5 mg/kg b.w.) + CFZ (1 mg/kg b.w.)]. Similarly, xenograft development and mice distribution were done using H23 isogenic resistant lines. The mice for all the groups were treated for 16 weeks, and the survival and tumor growth were assessed for 120 days.

Next, we also performed the drug combination study of adagrasib or sotorasib in combination with CFZ using the SW1573-derived xenografts in the athymic nude nu/nu mice. Thirty 8-week-old mice were divided into six groups of five animals [treatment with: (i) corn oil, i.e., vehicle control; (ii) sotorasib (10 mg/kg b.w.); (iii) CFZ (2 mg/kg b.w.); (iv) adagrasib (10 mg/kg b.w.); (v) sotorasib + CFZ; and (vi) adagrasib + CFZ. Treatment was started 12 days after the SW1573 NSCLC cells implantation to discern palpable tumor growth. Treatments were given to mice via oral gavage twice a week for 8 weeks. The changes in tumor area were measured for 8 weeks, and tumor weight and mice weight were measured at the end of the study. The tumor weights were compared between groups using an unpaired Student’s *t* test. A portion of the tumor was fixed in 10% buffered formaldehyde solution and paraffin-embedded for immunostaining or was snap-frozen in liquid nitrogen for further molecular analysis such as immunoblotting.

### Cell cycle analysis

We seeded 10,000 cells of SW1573 or H23 cells in a 96-well plate after 12 hours of seeding. A total of 50 μl of IncuCyte Cell Cycle Green/Red Lentivirus was added, and a stable cell line was generated according to the manufacture’s protocol (IncuCyte Cell Cycle Red/Green Lentivirus, #4779). The stable cell line expressing red fluorescence represents G_1_, green fluorescence represents S-G_2_, and yellow cells represents in transition from G_1_ to S while nonfluorescent cells are moving from M to G_1_. The stable cell lines were treated with KRAS inhibitors for 72 hours, and the cell cycle state was determined using the Attune NxT Cytometer.

### Immunofluorescence

SW15743 cells were seeded (50,000 cells per well) on each chamber of the Lab-Tek II chamber slide system (four-well format, catalog no. 12-565-7, Thermo Fisher Scientific). After 12 hours of cell seeding, the cell medium of each chamber was replaced with the fresh medium for the untreated chamber and adagrasib medium for the treated chambers (0.5, 1, or 2 μM). The cells were allowed to grow for 72 hours, followed by fixation with 4% formaldehyde for 30 min at room temperature, and blocked using the blocking buffer (catalog no. 12411, Cell Signaling Technology). Primary antibody against WNT2 (1:400 dilution; catalog no. 66656-1-Ig, Proteintech, USA) and phospho-S675–β-catenin (1:100 dilution; catalog no. 4176, Cell Signaling Technology, USA) were used for overnight staining at 4°C. The slides were washed, and secondary antibody was incubated for 2 hours followed by washing at room temperature. ProLong Antifade 4′,6-diamidino-2-phenylindole mounting medium (catalog no. 8961, Cell Signaling Technology, USA) was used for mounting the coverslips and imaged with Zeiss LSM 880 confocal microscope at the Light Microscopy/Digital Imaging Core Facility at City of Hope.

### Ethical approval

No human subjects were involved in the present study. All animal studies were conducted according to a protocol approved by the City of Hope Animal Care and Ethics Committee (IACUC, #16004). Any mice showing signs of distress, pain, or suffering due to tumor burden were humanely euthanized. All zebrafish experiments were carried out in accordance with a protocol approved by the IACUC (#18119).
