# Supplementary material for: Acquired resistance to KRAS G12C small-molecule inhibitors via genetic/nongenetic mechanisms in lung cancer
Source: Sci Adv. 2023 Oct 13;9(41):eade3816. doi: 10.1126/sciadv.ade3816 (PMC10575592; doi:10.1126/sciadv.ade3816)
Supplement: Supplementary file 1 — Supplementary Text Figs. S1 to S10 Tables S1 to S4 [file sciadv.ade3816_sm.pdf]

Science Advances
AAAS

Supplementary Materials for

Acquired resistance to KRAS G12C small-molecule inhibitors via genetic/nongenetic mechanisms in lung cancer

Atish Mohanty *et al.*

Corresponding author: Ravi Salgia, rsalgia@coh.org

Sci. Adv. **9**, eade3816 (2023)
DOI: 10.1126/sciadv.ade3816

The PDF file includes:

Supplementary Text
Figs. S1 to S10
Tables S1 to S4

Other Supplementary Material for this manuscript includes the following:

Data files S1 to S3

Supplementary Text

1. Homozygous KRAS mutant cells are more resistant to sotorasib than heterozygous cells.

Fold change in the cell growth after 72 h of drug treatment was calculated using a live cell imaging system. The percentage change in growth compared to untreated cells was used to determine the growth curves and IC50 value of sotorasib for all three cell lines (**fig. S1A**). For the spheroid growth assays, we also measured the red intensity with the passage of time as an additional readout of spheroid viability and observed a ~80% drop in red intensity with 0.3µM of sotorasib (**fig. S1B, green bar graph**). Further, we observed >8-fold increase in caspase activity over time at all concentrations of sotorasib for the H358 cell line (**fig. S1C, green line graph**). Similarly, for H23 cells, the red fluorescence intensity decreased by 40% for 0.3µM and further decreased by 74% at a concentration of 10µM sotorasib (**fig. S1B, blue bar graph**). The caspase activity increased by >5-fold for all the concentrations of sotorasib by 72 h (**fig. S1C, blue line graph**). Next, we measured the red intensity of SW1573 cells treated with sotorasib. We observed no change at 0.3 µM of sotorasib but, at higher doses the spheroid intensity dropped in a dose dependent manner, reaching a decrease of 70% at 10 µM (**fig. S1B, red bar graph**). Interestingly, the SW1573 spheroids did not show >2-fold increase in caspase activity despite the significant drop in the spheroid area and red intensity, suggesting that sotorasib suppresses SW1573 spheroid growth but could not induce cell death (**fig. S1C, red line graph**). Together, both the 2D and 3D data suggest that homozygous KRAS mutant SW1573 cells are least responsive compared to the heterozygous H358 and H23 cells.

2. *Homozygous KRAS mutant cells are more resistant to ARS1620 than heterozygous cells.*

We also tested the efficacy of the covalent inhibitor ARS1620 on these three cell lines. H358 cell growth was 40% inhibited by 1.2 µM of ARS1620 and further increasing the dose to 2.5, 5, or 10 µM induced 50% inhibition (**fig. S2A, green line graph**). Similarly, for the H23 cells, cell growth was inhibited by 30% at 2.5 µM concentration and inhibition was increased to 50% or 80% at higher concentrations of 5 µM or 10 µM, respectively (**fig. S2A, blue line graph**). Further, for the SW1573 cell line, ARS1620 could inhibit 20% of cell growth at a maximum concentration of 10 µM (**fig. S2A, red line graph**). The IC50 for H358 was calculated to be 0.5 µM, H23 to be 7.6 µM and for SW1573 it was greater than 10 µM (**fig. S2B**). ARS1620 inhibition assay on cell line-derived spheroids was also performed on these cell lines (**fig. S2C**). ARS1620 inhibited the spheroid area of H358 and H23 cell line derived spheroids by 70% at 10 µM concentration, whereas approximately 25% reduction was observed for of spheroid area of SW1573 cell line derived spheroids (**fig. S2D**). Similarly, the analysis of mean red intensity showed a minimum concentration of 5µM of ARS1620 could reduce H358 spheroids red intensity by 80%, H23 spheroids by 50%, and the SW1573 spheroids by 40% (**fig. S2E**). Finally, in the live cell caspase-3/7 assay for the spheroids, a 19-fold increase in activity was

observed for the H358 cell line derived spheroids within 24 h of 2.5 µM ARS1620 treatment, whereas there was only a 5-fold increase for the H23 and no change for SW1573 cell line derived spheroids (**fig. S2F**). Thus, the data concludes that the H358 cell line is highly sensitive to ARS1620 followed by H23, and the SW1573 cell line is the least sensitive to these covalent inhibitors. The sensitivity pattern of the cell lines remains similar to that of the sotorasib treatment.

3. *Sotorasib or ARS1620 treatment disrupts the ITGB4/PXN axis and activates AKT signaling.*

Immunoblotting analysis post-sotorasib or ARS1620 treatment was repeated with the IC50 concentration of the respective drugs for the specific cell lines used. The KRAS is known to interact with PI3K and AKT which are required for PI3K-AKT- mTOR signaling (**fig. S3A**). Immunoblotting experiments showed a decrease in the expression of phospho-S473 AKT, phospho-RB, ITGB4, and PXN and a simultaneous increase in the expression of cleaved PARP, γH2AX, and p27 for the sensitive lines (H358 and H23), (**fig. S3B**). Interestingly, the ERK phosphorylation did not show any major changes and AKT got hyper phosphorylated in the SW1573 Cell line. The data is indicative that in the resistant line, there is an activation of alternative pathways to compensate for KRAS inhibition. A similar experiment post-ARS1620 treatment showed a significant reduction in the expression of ITGB4 but no changes in the expression of PXN and FAK. Again, for the sensitive line H358, there was a reduction in the expression of phospho-AKT, ERK, Rb, and an increase in the levels of cleaved PARP and γH2AX expression. The H23 cells, which were sensitive to sotorasib, could tolerate ARS1620 treatment and did not show any significant change. Likewise, the SW1573 cells remain unaffected (**fig. S3C**). Thus, the data suggest a possible role of ITGB4 expression and AKT phosphorylation in developing tolerance to KRAS inhibitors.

4. *Isogenic KRAS resistant cell development and ITGB4 correlation.*

We treated the H358, H23, and SW1573 cells continuously with sotorasib to generate tolerant cell lines that could proliferate even in the IC50 concentration of sotorasib (**fig. S3D**). The immunoblotting analysis of the lysate obtained from the parental and resistant H23 or SW1573 cell lines showed increased expression of ITGB4, whereas the H358 cell line showed decreased expression of ITGB4. These H23 resistant cells upon continuous treatment could become resistant to up to 20µM of sotorasib. However, the same technique of generating highly resistant cell lines was not successful for the H358 cells and that correlated with the reduction in ITGB4 expression (**fig. S3D**).

5. *Knocking down ITGB4 and PXN inhibited cell growth in the presence of sotorasib.*

To validate the role of ITGB4 and PXN in sotorasib resistance, we knocked down ITGB4, PXN, or both ITGB4/PXN in the 3 cell lines using siRNA. At 24 h post-transfection, the cells were split into two groups. The first group was untreated while the second group was treated with each cell line's specific IC50 concentration of sotorasib. Cell proliferation was measured in real time using IncuCyte. In the untreated condition, we observed partial

inhibition in proliferation for all 3 cell lines upon knocking down both ITGB4 and PXN. However, sotorasib presence had a significant inhibitory effect on the ITGB4 knockdown or ITGB4/PXN double knockdown cells but not cells with PXN knockdown alone (**fig. S3E**).

6. *CFZ and sotorasib combination induce the synergistic effect.*

The inhibition matrix of CFZ and sotorasib was used to identify the drug synergy as mentioned in **Fig. 2E**. The synergy score for different concentrations of the drugs is represented as a 2D and 3D contour plot (**fig. S4A and B**). The drug combination experiment was repeated; however, the CFZ concentration was kept constant (9.5 nM) and the concentration of sotorasib increased from 1 µM to 64 µM. The combined inhibitory effect of CFZ (9.5nM) and sotorasib (1-8 µM) was lesser compared to CFZ alone, indicating an antagonistic effect of the drug combination at lower concentrations of sotorasib. The same concentration of CFZ together with 16 or 32 µM of sotorasib induced a synergistic effect (the combined drug inhibition is more than the inhibition by sotorasib alone and CFZ alone) **(Fig. 2F, fig. S4C).** The experiments were repeated in hypoxic conditions by culturing the cells in 5% of oxygen. Synergistic effect was observed in the combination of CFZ (9.5 nM) with sotorasib (16 µM), similar to normoxia. However, in hypoxia, the single drug effect of CFZ was stronger than in the normoxia condition (**fig. S4D**). We then measured the effect on cell proliferation using a single dose of CFZ (10 nM) and increasing concentrations of sotorasib (8, 16, or 32 µM), and the data confirmed a significant reduction of cell growth in combination compared to the single drug alone (**fig. S4E**). Like SW1573, the drug combination was also tested in the H23 cells and found to be effective. A reduction in the expression of ITGB4, phospho-AKT, phospho-ERK, CCND1, and upregulation of cleaved γH2AX was observed in the immunoblots of H23 cells treated with drug combination also **(fig. S4F**). Thus, these results suggest that CFZ can potentially be used as a small molecule for inhibiting ITGB4, AKT-mTOR signaling, and sensitizing sotorasib refractory tumors to sotorasib treatment.

7. *Upregulation of WNT2 genes in an isogenic sotorasib resistant cell line.*

Differential expression analysis suggests significant changes in the expression of 712 genes and the clustering analysis clearly shows the changes in the gene expression upon sotorasib treatment **(fig. S5A)**. Two strategies were used to calculate gene set enrichment, one strategy used Enrichr (93) to calculate the "Hallmark" genes curated from 2020 while the other strategy used Gene Set Enrichment Analysis (GSEA, version 2.2.2; MsigDB, version7.0) (57). We presented the Hallmark gene set enrichment analyzed using Enrichr for FDR < 0.0001 in **fig. S5B and C**. The Hallmark gene sets enrichment like TNF signaling via NF-kB, KRAS signaling up, hypoxia, and inflammatory response were consistently downregulated for both treatments whereas the EMT associated genes were part of both positive and negative enrichment classes (**fig. S5B and C, Excel Files S1-3**). The differentially expressed genes overlapping the Hallmark KRAS-UP gene set are presented as a heatmap (**fig. S5D**). Furthermore, the GSEA enrichment score for KRAS signaling upregulation was negative for both treatments (**fig. S5E**), but the

enrichment was only statistically significant for Parent Versus Control (Parent versus Control FDR = 0.0039, Resistant versus Control FDR = 0.62). The list of GSEA enriched pathways are presented in **Tables S1 to 4.**

RNA sequencing analysis, suggested upregulation of *CCL2, CFTR, and WNT2* by 86-fold, 51-fold, and 47-fold, respectively, in the resistant cell line. These three genes were selected for further validation by qPCR using TaqMan Probes. The results confirmed significant upregulation of these genes upon short-term exposure to sotorasib in parental cells, and multifold upregulation in the isogenic resistant cells upon sotorasib treatment (**fig. S5F**). Using a SYBR Green qPCR assay, we also validated the expression of the other genes (listed in **Fig. 3D**) compared to the untreated cells (**fig. S5G**). The lung cancer TCGA database revealed that WNT2 expression is lower in tumor tissue compared to normal tissue. However, some studies reported the aberrant activation of WNT2 autocrine signaling in ~50% of NSCLC primary tumors and cell lines. Therefore, the functional significance of the WNT signaling pathway in sotorasib resistance or sensitivity warrants further investigation. We treated the three cell lines with half IC50 and IC50 doses of sotorasib for 3 days, and immunoblotting was done. The blots showed increased protein expression of WNT2 in the H23 and H358 cell lines upon sotorasib treatment, whereas the SW1573 cells already had high expression of WNT2 (**fig. S5H**).

Next, we performed exome sequencing analysis on the H23 cells resistant to 7.5 µM of sotorasib (Iso 7.5 cells). The mutational analysis did not show any additional mutations in the KRAS gene, but several nonsynonymous mutations were observed in the genes encoding *PCMI, CNTNAP3, WDR64, PCSK4, and TAS1R3* in the resistant cell lines (**fig. S5I**). Subsequently, we continued to treat these Iso-7.5 resistant cells with increasing concentrations of sotorasib until they became resistant to 20µM sotorasib (Iso 20 cells). The genomic mutational analysis of these Iso-20 cells showed new nonsynonymous mutations in genes *LMO2, SIX5, SOX2, PRICKLE2, and ARHGAp33*, which were not present in the Iso-7.5 cells (**fig. S5I**). Interestingly in the RNA seq analysis, we did not observe any significant change in the expression of these mutated genes suggesting a nonsignificant contribution of these genes in sotorasib resistance.

8. WNT2 knockout sensitizes cells to sotorasib.

Using a CRISPR/Cas9 plasmid against the WNT2 locus, we generated WNT2 knockout SW1573 and H23 cell lines and determined their sensitivity to sotorasib by live cell proliferation assays. We observed reduced expression of WNT2 in the puromycin-selected H23 cells, suggesting a majority of cells have the knockout (**fig. S6A, immunoblot**). Nonetheless, we observed that WNT2 knockout alone was enough to inhibit cell proliferation in H23 cells, and the addition of sotorasib further suppressed cell growth (**fig. S6A, line graph**). However, in SW1573 cells we did not observe any significant change in cell growth compared to the control both in the absence or presence of sotorasib (**fig. S6B, line graph**). The SW1573 cells used in the assay showed approximately 50% reduction in WNT2 expression indicating a mixed population of knockout and non-knockout cells or a heterozygous deletion (**fig. S6B, immunoblot**).

Interestingly, another possibility of a lesser effect of WNT2 KO in the SW1573 cell line is the presence of CTNNB1 mutation, which can induce its *de novo* activation and stability.

Next, we tested whether knocking down ITGB4 in the WNT2 knockout H23 cells would further sensitize them to sotorasib. We transfected the H23 WNT2 knockout cells with an ITGB4 siRNA followed by treatment with the IC50 concentration of sotorasib and discerned the effect on cell proliferation in real time. Indeed, ITGB4 knockdown further inhibited cell growth upon the addition of sotorasib (**fig. S6C**). Thus, the data indicate that both the ITGB4 and WNT2 signaling work independently and inhibiting both genes could reduce drug resistance significantly.

9. Knocking down CTNNB1 and ITGB4 sensitize sotorasib resistant cells to sotorasib.

To decipher the involvement of WNT2/β-catenin signaling in imbibing sotorasib resistance, we transfected the H23 parental cell line, sotorasib resistant (20 µM) isogenic line as well as SW1573 cells with 10 nM of CTNNB1 siRNA, 10 nM ITGB4 siRNA, or with both CTNNB1 (10 nM) and ITGB4 (10 nM) siRNA. Their proliferation in response to sotorasib treatment was followed for 96 hours, which reveals that sotorasib treatment could inhibit H23 or H23 Iso 20 cell proliferation significantly upon knocking down both ITGB4 and CTNNB1 **(fig S6D and E).** The knockdown of CTNNB1 in SW1573 cells followed by sotorasib treatment also inhibited cell growth significantly **(fig S6F)**. The results were suggestive that WNT2/β−catenin signaling was contributing to sotorasib resistance and targeting the signaling could reverse tolerance to sotorasib.

10. β–catenin inhibitor (BC2059) and sotorasib are synergistic

To determine the effect of a β-catenin-specific inhibitor, BC2059, against the resistant SW1573 cell line, we treated the cells with varying concentrations of sotorasib and BC2059. The inhibition matrix showed that 10 nM of BC2059 could inhibit SW1573 cell growth by 10%, whereas a combination of 10 nM of BC2059 with 1 µM, 16 µM, or 24 µM of sotorasib could inhibit cell growth by 24%, 33%, or 49%, respectively (**fig. S7A**). The combinations of 10 nM BC2059 and 1 µM, 16 µM, or 24 µM of sotorasib had a synergistic effect as indicated by the synergy heatmap with a synergy score of 15.54 and the 3D contour plot (**fig. S7B to D**). The results suggest that combining BC2059 and sotorasib could be a good therapeutic strategy for suppressing the growth of sotorasib resistant tumors. We then analyzed changes in protein expression and signaling in response to increasing concentrations of BC2059 alone but observed no significant changes in the expression of WNT2, β-catenin, or ITGB4 (**fig. S7E**), indicating that BC2059 may affect the localization of β-catenin or disrupt its protein-protein interactions, which would require further investigation. To ascertain the effect of drug combination on the SW1573 cells, we treated the cells with increasing concentrations of sotorasib alone or in combination with BC2059 for 72 h, and changes in protein expression and signaling were determined by immunoblotting (**fig. S7F**).

11. *Sotorasib resistant cells are sensitive to adagrasib.*

The cell proliferation assay with increasing concentrations of adagrasib was also tested using the H358 and H23 cell lines. The data indicated that adagrasib could suppress the growth of cells significantly and the IC50 for the two cell lines was calculated to be 0.51 µM, and 2.15 µM, respectively (**fig. S8A**). The signaling changes induced by adagrasib were also analyzed and are shown in **fig. S8B**. Having observed that sotorasib refractory cells can be sensitive to adagrasib in 2D cultures, we then evaluated the efficacy of adagrasib in 3D spheroid cultures by determining the changes in spheroid growth and caspase activity (**fig. S8C**). The changes in spheroid growth were analyzed upon adagrasib treatment (10µM). The spheroid growth was completely abrogated within 48 h for H358 and by 96 h for the H23 spheroids, which was not the case when spheroids derived from the same cells were treated with sotorasib. Reducing the concentration to 5µM also abrogated spheroid growth for the H358 and H23 spheroids (**fig. S8D**). The red mean intensity changes for the final time point were analyzed and found to be 80% reduced at a minimum concentration of 0.6 µM of adagrasib for H358 cells, whereas for the H23 spheroids, the reduction was 40% (**fig. S8E**). The loss of red intensity also indicates the reduction in the spheroid viability, which was confirmed by the caspase assay. The caspase-3/7 activity was upregulated within 24 h of 10µM adagrasib treatment in H358 and H23 spheroids, and for the lower concentration, the caspase activity was upregulated by 48h (**fig. S8F**). Together, these data demonstrate significant differences in the signaling alterations induced by the two KRAS G12C inhibitors and show that adagrasib treatment exhibits a more robust phenotype compared to sotorasib.

12. *Knocking down WNT2 or inhibiting ITGB4 and WNT2/β-catenin signaling sensitizes cells to adagrasib.*

We used the WNT2 KO H23 and SW1573 cell lines to determine their sensitivity to 4 days of treatment with adagrasib. The data revealed a 4-fold increase in cell growth for the control cells and a 3-fold increase for the cells treated with 0.25 µM adagrasib. WNT2 KO cells had a weaker proliferation with only a 2.5-fold increase in growth in the absence of adagrasib, whereas, in the presence of 0.25 µM adagrasib, we only observed a 1.5fold increase in cell count, suggesting that adagrasib treatment of WNT KO H23 cells exhibited a stronger inhibition of cell proliferation (**fig. S8G, left graph**). In contrast, WNT2 KO in the SW1573 cell line did not sensitize the cells to adagrasib induced growth inhibition (**fig. S8G, right graph**).

Next, we screened for the inhibitory effect of adagrasib and CFZ drug combination against the SW1573 cells. We observed that after 72 h of drug treatment,10 nM of CFZ was able to inhibit the growth of the SW1573 cells by 60%, and the addition of 1 µM of adagrasib had an additional effect leading to 75% inhibition compared to 47% inhibition by adagrasib alone. A similar effect was also observed for the combination of 1µM adagrasib and 5.5 nM of CFZ in the H23 cell line (**fig. S8H**). The data suggests the inhibitory effect of adagrasib and CFZ drug combination to be additive. We also validated the inhibitory effect of adagrasib on sotorasib refractory cells by determining its effect on the isogenic H23 sotorasib resistant line (resistant to 20 µM sotorasib). This analysis clearly showed that

adagrasib could significantly inhibit the growth of these cells even at lower concentrations (**fig. S8I**), suggesting that adagrasib monotherapy remains active even in conditions of sotorasib resistance, and in combination with CFZ can induce even stronger inhibition.

13. Gating strategy for Cell-Cycle analysis.

IncuCyte® Cell Cycle Green (GFP) /Red (mKate2) Lentivirus was used to infect the cell lines and these infected cells were selected against the antibiotic (1 µg/ml) puromycin for generating stable cell lines. The selected cells were cultured as well as stocked for experimental purposes. For the FACS experiment, the cells were seeded at a density between 100,000 cells / well of 6 well culture plates and after 12hr of seeding, the old media was replenished with fresh media or with media containing specific inhibitors. After 72 h of treatment, the floating as well as adherent cells were harvested, washed with PBS two times, and resuspended in the 500µl of PBS. These resuspended cells were used for FACS using the Attune Nxt Flow cytometer and the software Attune NxT Software. FlowJo V10 was used for the gating and analysis as shown in the **fig S9.** The IncuCyte cell cycle markers are nuclear specific; therefore, we used the stable cell lines expressing nuclear specific green or red fluorescent proteins for compensation. SSC-H /FSC-H gates were used to gate the cells and these gated cells were used to gate the singlets using the SSC-H /SSC-A gates. The singlets were used to determine DAPI negative versus positive cells (**fig. S9A and B**). The DAPI negative cells were used to analyze the green only (BL1: excitation 488nm, emission filter 530/30, Y-axis), red only (YL2: excitation 561nm, emission filter 620/15, X-axis), red and green or none of the fluorescent protein expressing cells. The SW1573 regular cell line was used as a negative control to identify background fluorescence and gate the negative population **(fig. S9A).** The untreated SW1573 cells expressing the cell cycle fluorescent markers were used for the gating for positive population green, red, and both red and green fluorescence (**fig. S9B**).

14. In vivo studies of the sotorasib or adagrasib combination with CFZ.

Mice xenografts were created with SW1573 cells to determine the antitumor effects of sotorasib, adagrasib, and CFZ, and their combinations *in vivo* (**fig. S10A**). Each mouse was injected with 1 million SW1573 cells diluted in 100µl of saline. Once a palpable tumor formed, the mice were divided into groups, and drug treatment was initiated. The changes in tumor growth were also measured over time. Next, we also analyzed the changes in mice body weight over time and did not observe any significant reduction in the mice weight, suggesting the drug combination was not toxic to the mice (**fig. S10B**). Compared to the control group, adagrasib, sotorasib, and CFZ treatment caused a significant reduction in tumor weight measured at the end of the study (day 56), and combining CFZ with adagrasib or sotorasib led to a significant reduction in tumor weight compared to single treatments (**fig. S10C**). Treatment with sotorasib, adagrasib, or CFZ alone caused a ~52%, 58%, and ~56% reduction in the tumor area, respectively. Combinatorial treatments showed reductions of ~65% with the sotorasib plus CFZ combination and ~69% with the adagrasib plus CFZ combination (**fig. S10D**). No macroscopic evidence of metastasis to other organs was evident for any experimental groups. Together, these

results underscore the potential of sotorasib or adagrasib plus CFZ combination therapy as a novel therapeutic strategy for NSCLC, especially in sotorasib resistant lung cancer.

Supplementary Excel Files:

Excel File 1: Differential gene expression tables.

Excel File 2: Differential pathway enrichment (Downregulated),

Excel File 3: Differential pathway enrichment (Upregulated),

Supplementary Figure 1

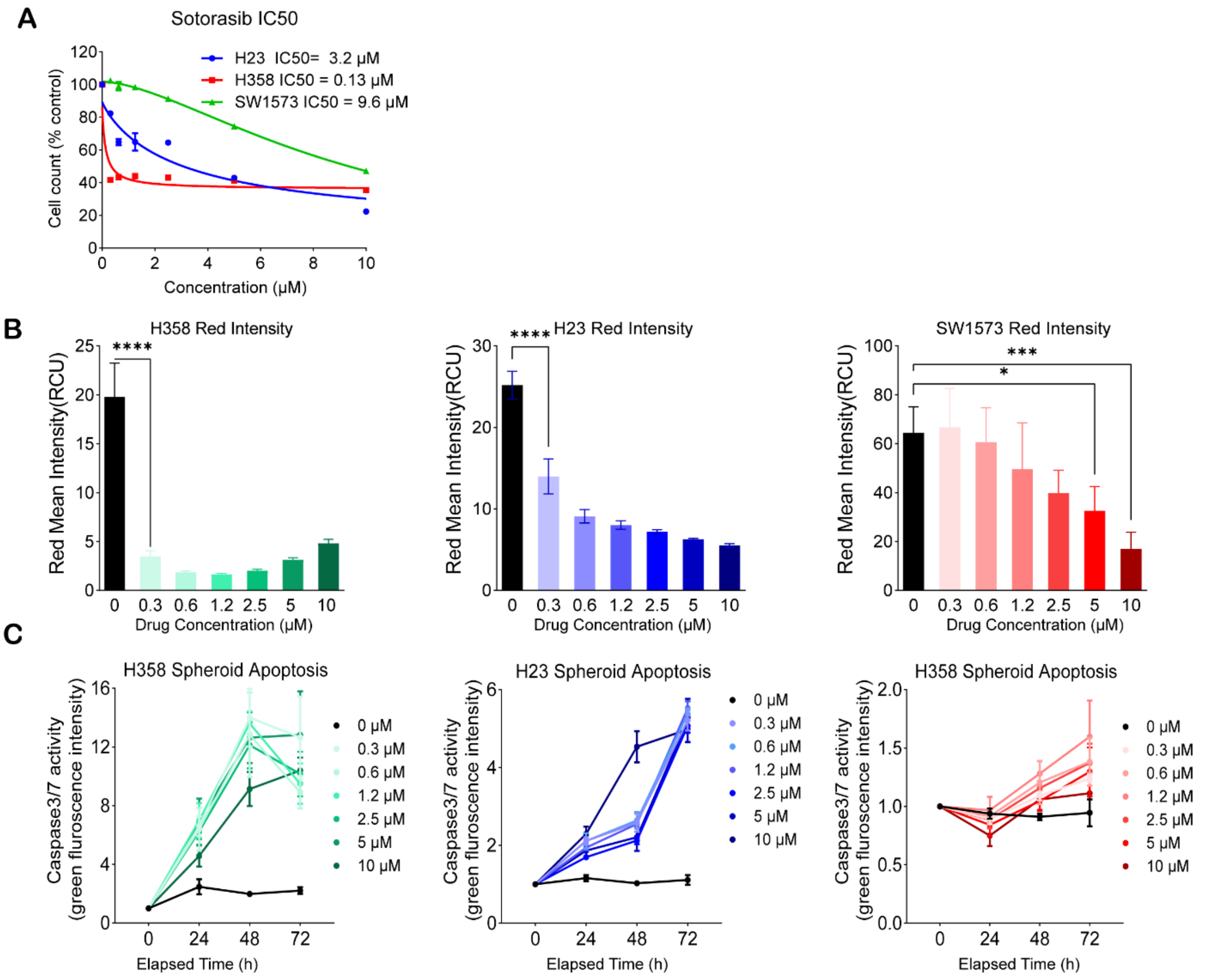

Supplementary Figure 1. NSCLC KRAS G12C cell lines respond to KRAS G12C inhibitor sotorasib at varying concentrations. **A)** Sotorasib (AMG510) IC50 values for H23, H358, and SW1573 cell lines at 72 h. The IC50 calculation was done using the nonlinear regression analysis, sample per group n=3. **B)** Mean red intensity analysis showed a significant reduction in the mean intensity at 0.3µM of sotorasib for H23 and H358 cell line derived spheroids. A similar effect was seen in SW1573 cells at a higher concentration of 10 µM. Ordinary one-way ANOVA was used to calculate the statistical significance, sample per group n=4, *p<0.01, ***p<0.001, ****p<0.0001. **C)** The analysis of caspase3/7 activity showed an 8-12 fold increase in caspase activity within 72 h of sotorasib treatment for the H358 cells whereas there was only a 5 to 6-fold increase for the H23 cells, and less than 2 fold change for the SW1573 cells. Two-way ANOVA test was used to calculate the statistical significance., sample per group, n=4.

Supplementary Figure 2

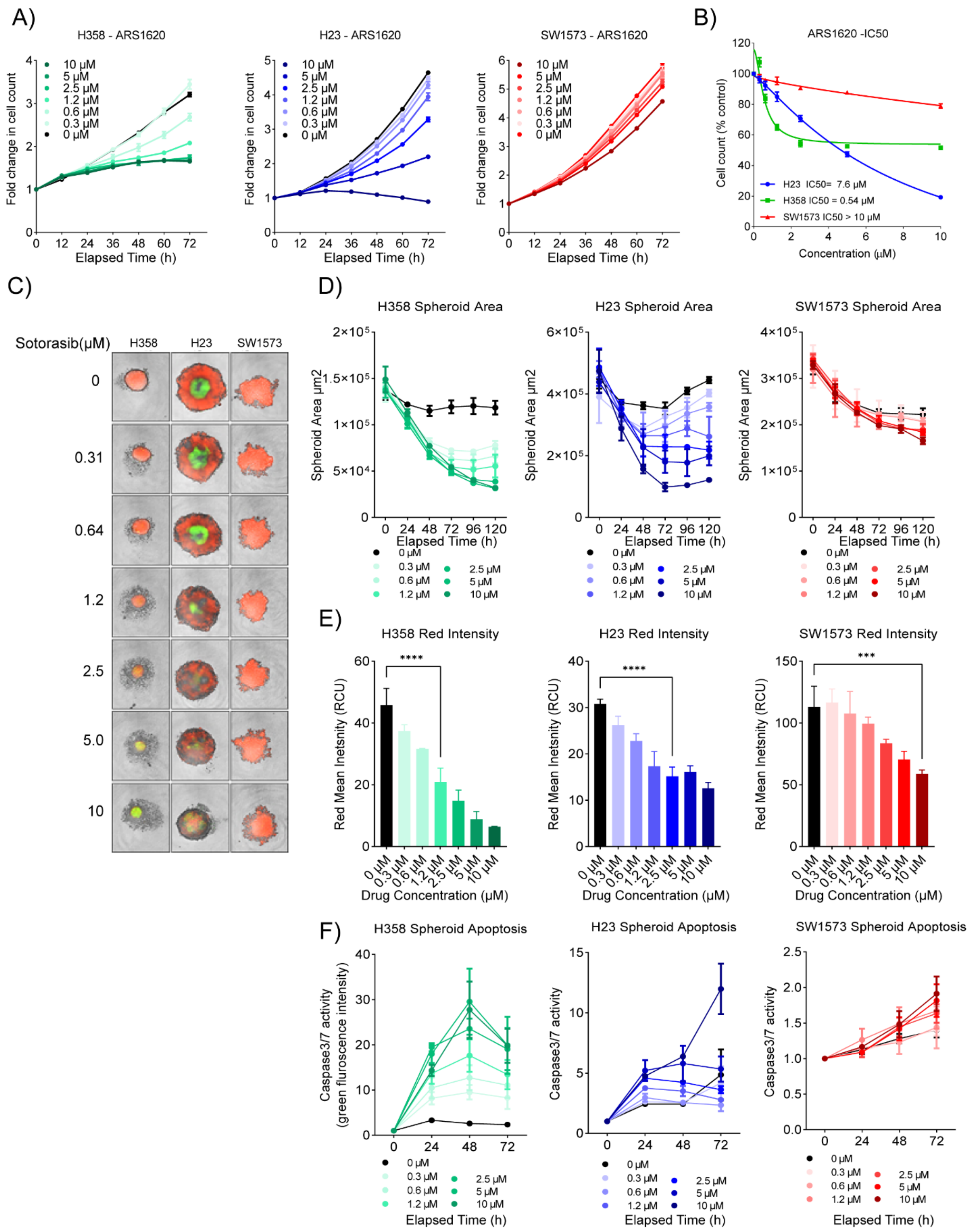

Supplemental Figure 2. NSCLC KRAS G12C cell lines respond to KRAS G12C inhibitor ARS1620 at varying concentrations**. A)** H358, H23, and SW1573 were treated with an increasing concentration (0.3-10 µM) of ARS1620, and fold change in cell count was determined over the course of 72 h. Two-way ANOVA test was used to calculate the statistical significance., sample per group, n=3**. B**) ARS1620 IC50 values for H23, H358, and SW1573 cell lines at 72 h. The IC50 calculation was done using the nonlinear regression analysis, sample per group n=3. **C)** H358, H23, and SW1573 cell line-derived spheroids were treated with an increasing concentration (0.3-10 µM) of ARS1620, and images were taken with IncuCyte live cell imaging system on day 5. Red fluorescence indicates cell viability and green fluorescence indicates caspase-3/7 activity. **D)** Spheroid area and growth kinetics data indicated a 70% reduction in spheroid growth at 10µM of ARS1620 for both H358 and H23 cell lines, and a ~25% reduction in the SW1573 cell line. Two-way ANOVA test was used to calculate the statistical significance, samples per group n=4. **E)** Mean red intensity analysis showed 5µM of ARS1620 could reduce H358 spheroid red intensity by 80%, H23 spheroids by 50 %, and the SW1573 spheroids by 40 %. Ordinary one-way ANOVA was used to calculate the statistical significance, sample per group n=4, ****p<0.0001. **F)** The analysis of caspase3/7 activity showed a 19-fold increase in activity within 24 h of 2.5 µM of ARS1620 treatment for the H358 cells whereas there was only a 5-fold increase for the H23 cells, and no change in the SW1573 cells. Two-way ANOVA test was used to calculate the statistical significance, samples per group n=4.

Supplementary Figure 3

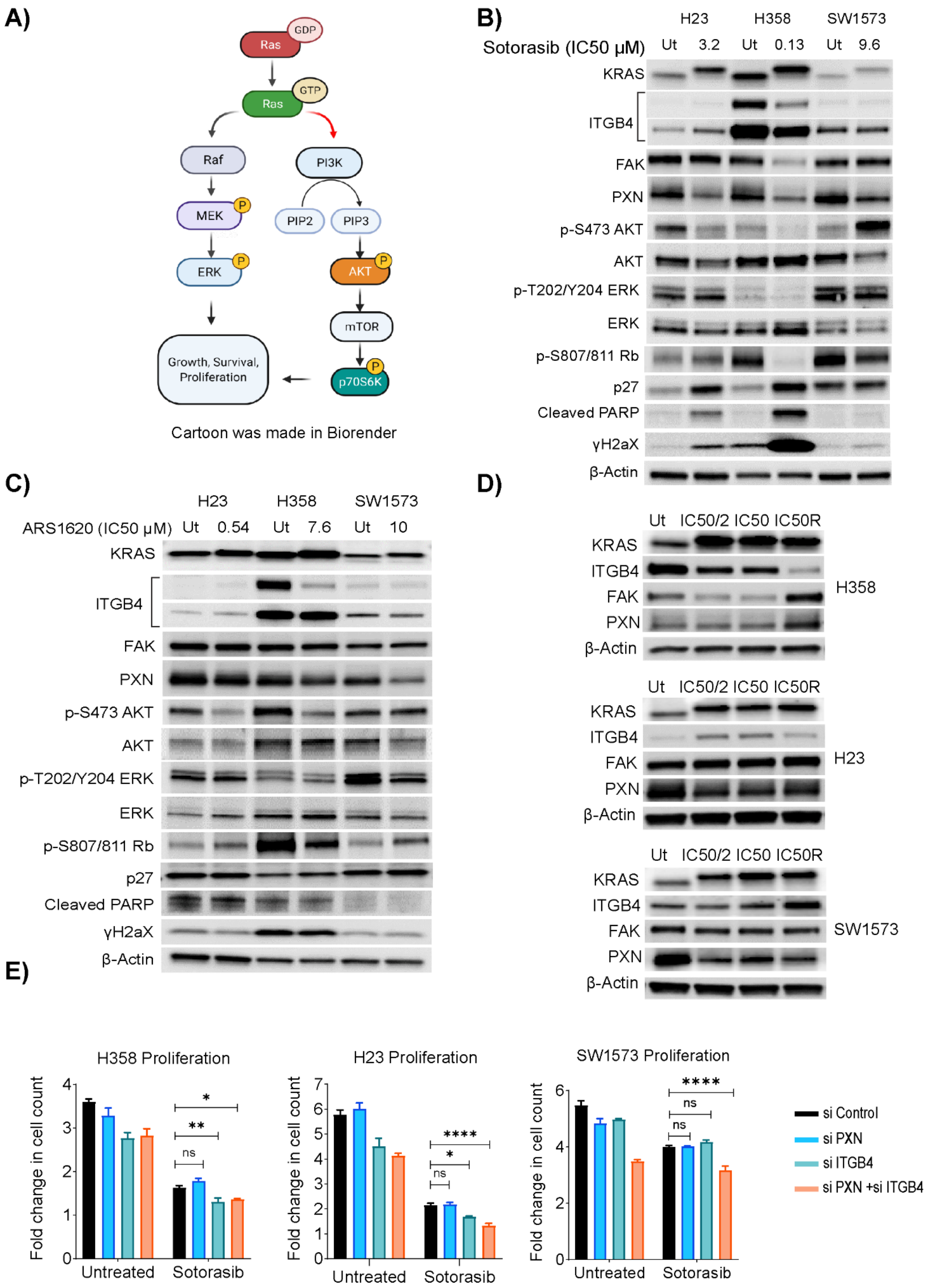

Supplementary Figure 3. Supporting data for Figure 2. **A)** A cartoon showing the Ras signaling through ERK and mTOR, made using BioRender. **B)** H23, H358, and SW1573 cells were treated with sotorasib IC50 concentration for 72 h, and changes in protein expression and signaling were determined by immunoblot. **C)** H23, H358, and SW1573 cells were treated with ARS1620 IC50 concentration for 72 h, and changes in protein expression and signaling were determined by immunoblot. **D)** H358, H23, and SW1573 cells were treated with half IC50 (IC50/2) and IC50 concentration of sotorasib for 72 h. These same cells were made resistant to IC50 concentration of sotorasib (IC50 R), and changes in protein expression were determined by immunoblot. **E)** Effect of PXN and ITGB4 single knockdowns and double knockdown with sotorasib on cell growth after 72 h of drug treatment. Two-way ANOVA was used to calculate the statistical significance for each group (si Control, si PXN, si ITGB4, and si PXN + si ITGB4, n = 3 per group, ns= not significant, *p<0.05, **p<0.001, ***p=0.0001, ****p<0.0001)

Supplementary Figure 4.

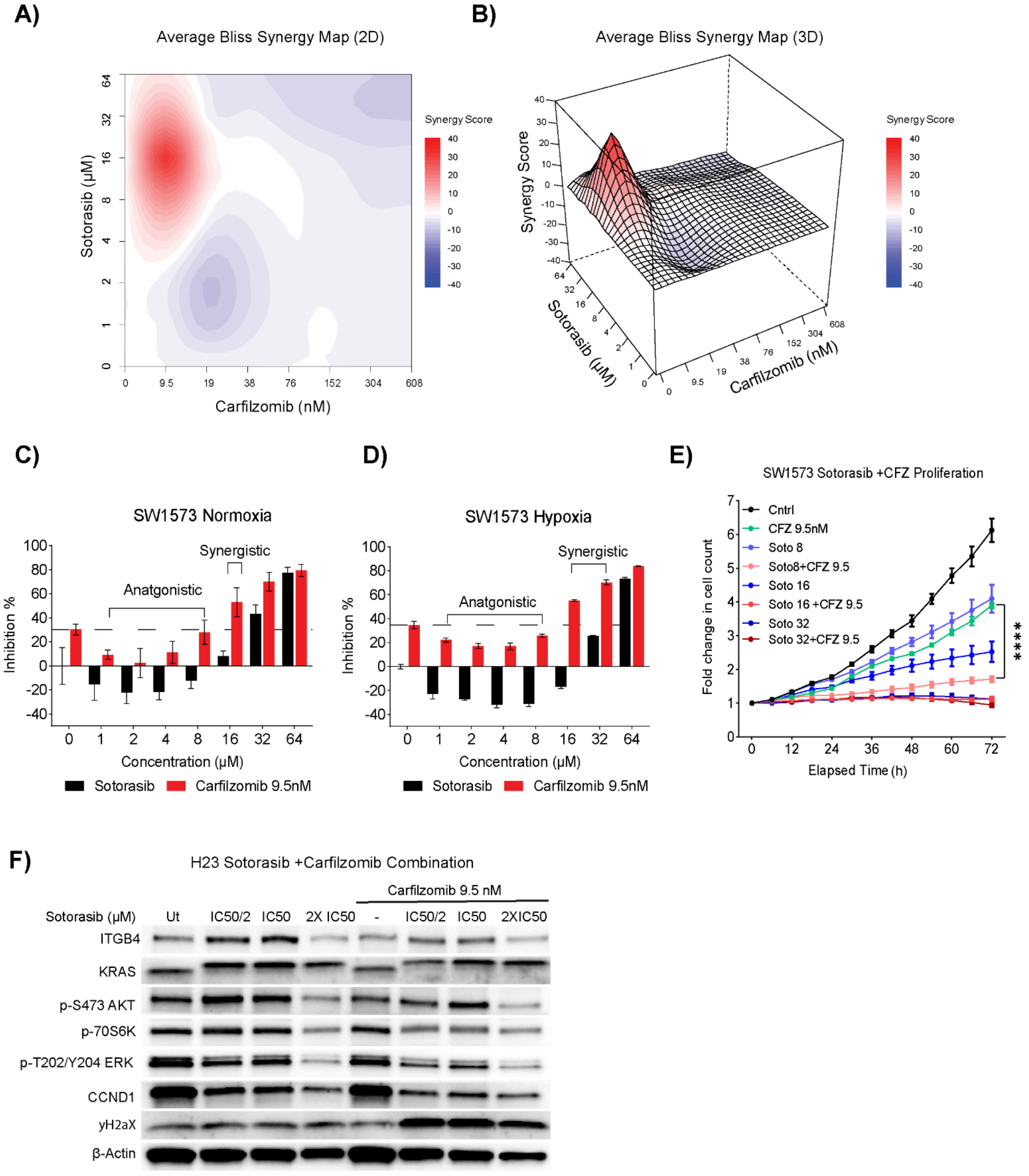

Supplementary Figure 4. Supporting data for Figure 2. **A)** Highest synergy score for sotorasib and CFZ was calculated for SW1573 cells and represented as 3D Bliss plot. **B and C)** Percent of inhibition in SW1573 cells was determined under normoxia and hypoxia culturing conditions with increasing concentrations of sotorasib (1-64 µM) and fixed concentration of CFZ (9.5 nM) to determine which are antagonistic, additive, and synergistic. **D**) SW1573 cells were treated with an increasing concentration of sotorasib (8-32 µM) with or without the addition of CFZ 9.5 nM and fold change in cell count was determined over the course of 72 h. Two-way ANOVA was used to calculate the statistical significance for each time point and for each drug concentration, n = 3 per group. **E and F**) The combination effect of CFZ and sotorasib on ITGB4, AKT, ERK activation, and γH2AX was analyzed using SW1573 and H23 cell line**,** respectively.

Supplementary Figure 5

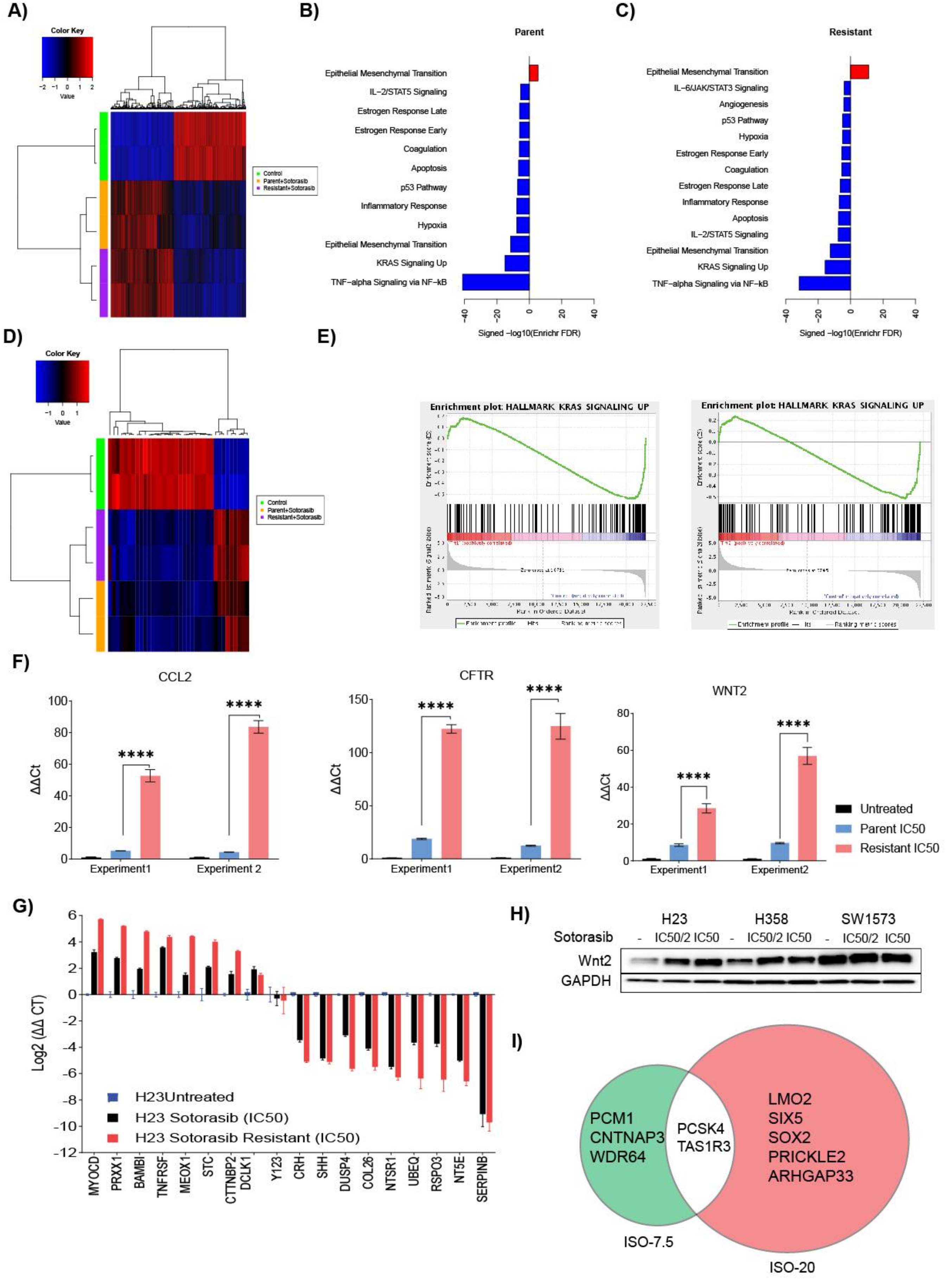

Supplementary Figure 5. Supporting data for Figure 3A) Clustering analysis of 712 genes that were downregulated or upregulated and common to the 3-day treatment group and the 7 weeks treatment group. **B** and **C**) Top Enrichr gene set enrichment analysis done on the gene expression data to find significant upregulation of EMT gene sets, with FDR < 0.0001. **D**) Heat map representing the differential changes in the expression of genes contributing to Hallmark pathway KRAS Signaling Upregulation. **E**) GSEA MSigDB Hallmark enrichment plots for KRAS-UP (top FDR = 0.0039, bottom FDR = 0.62. **F**) Taqman probe-based qPCR analysis to confirm upregulation of top 3 upregulated genes (CCL2, WNT2, CFTR) previously determined by RNA sequencing. Ordinary one-way ANOVA was used to calculate the statistical significance, sample per group n=4, ****p<0.0001. **G**) Using SYBR Green qPCR assay, RNA sequencing data was validated by confirming upregulation or downregulation of the other top 10 genes. **H**) H358, H23, and SW1573 cells treated with half IC50 dose and IC50 of sotorasib for 72 h confirm an increase in WNT2 protein expression. **I**) The mutations identified in exome sequencing analysis on the H23 isogenic cells resistant to 7.5 µM (Iso 7.5 cells) and 20 µM (Iso 20 cells) of sotorasib.

Supplementary Figure 6

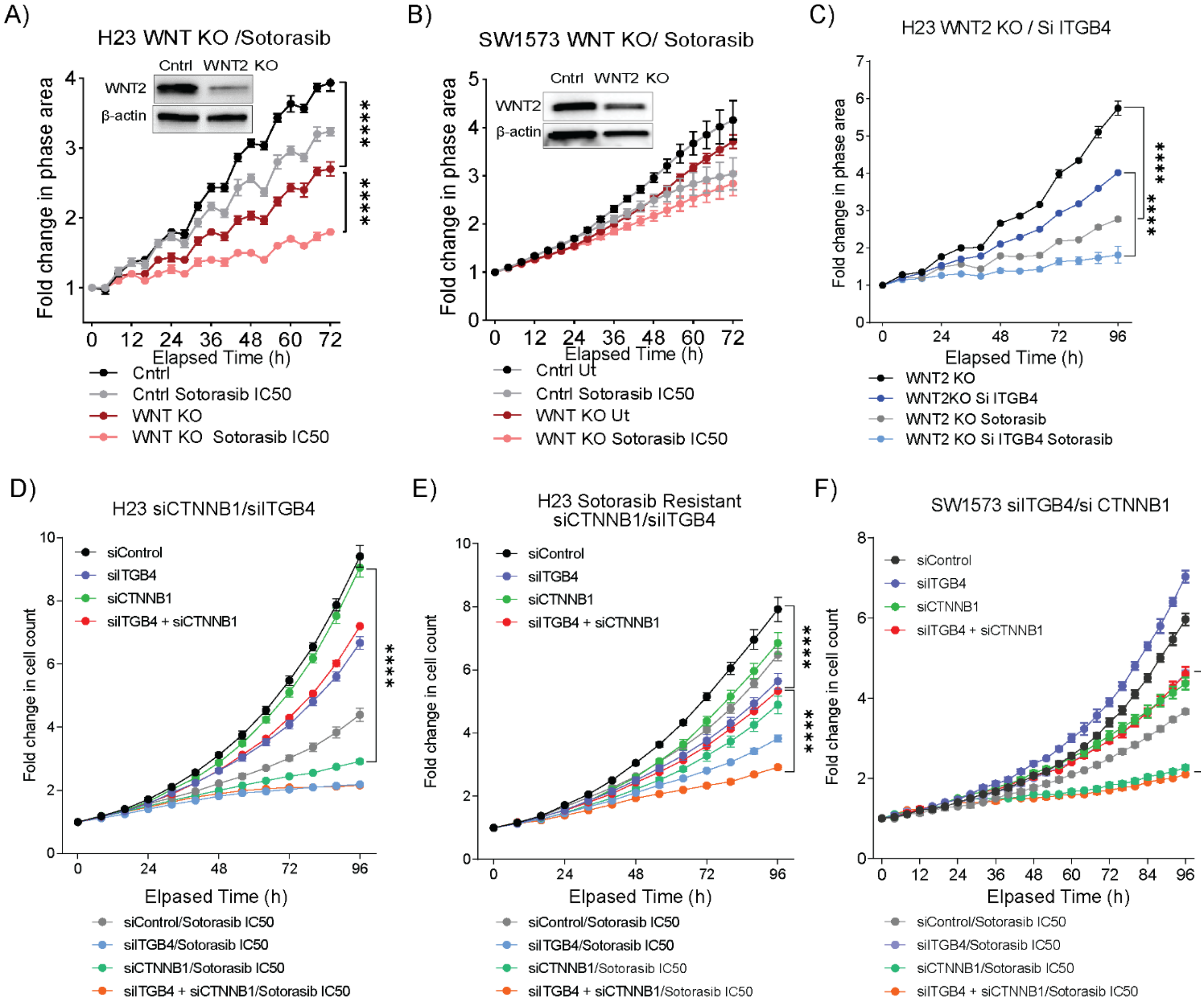

Supplementary Figure 6. Supporting data for Figure 3. **A and B)** WNT2 CRISPR knockout (KO) and addition of sotorasib IC50 concertation inhibiting proliferation of H23 and SW1573 cells, respectively over the course of 72. Immunoblot shows the knockout efficiency in mixed populations. Two-way ANOVA test was used to calculate the statistical significance, samples per group n=3, ****p<0.0001. **C)** ITGB4 knockdown in WNT2 KO H23 cells enhances the sensitivity of H23 to sotorasib. Two-way ANOVA test was used to calculate the statistical significance, samples per group n=3, ****p<0.0001. **D, E, and F**) Effect of ITGB4 and CTNNB1 single knockdowns and double knockdown with sotorasib 3 µM on the proliferation of H23 sotorasib (20 µM)-resistant cells over 96 h (line graph) and percent change in growth at 96 h (bar graph). Two-way ANOVA was used to calculate the statistical significance for each time point and for each condition (si Control, si ITGB4, si CTNNB1, si CTNNB1+si ITGB4, n = 3 per group, ****p<0.0001.

Supplementary Figure 7

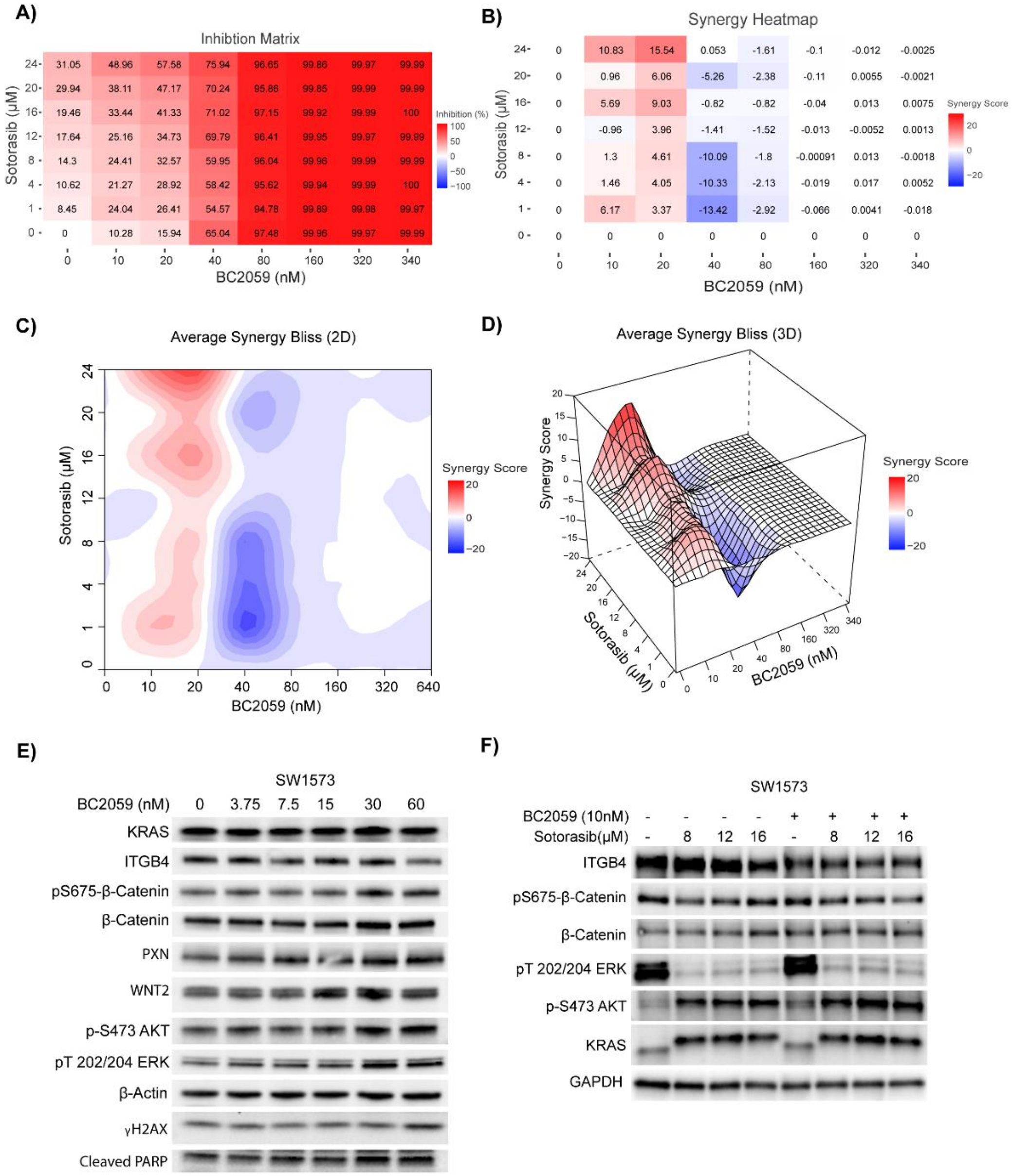

Supplementary Figure 7. Supporting data for Figure 3. **A**) SW1573 cells were treated with 8 different concentrations of sotorasib (AMG510) and β-catenin inhibitor BC2059 in the form of a matrix to determine % inhibition of proliferation. **B**) Synergy scores were calculated and represented as a Bliss plot. **C**) and **D**) The highest synergy score for sotorasib and BC2059 was calculated for SW1573 cells and represented as 2D and 3D average synergy Bliss plots. **E**) SW1573 cells were treated with an increasing concentration of sotorasib 0 to 60 nM for 72 h and immunoblotting was done to determine significant changes in protein expression. **F**) The SW1573 cells were treated with increasing concertation of sotorasib alone or in combination with BC2059 for 72 h changes in protein expression and signaling was determined by immunoblotting.

Supplementary Figure 8.

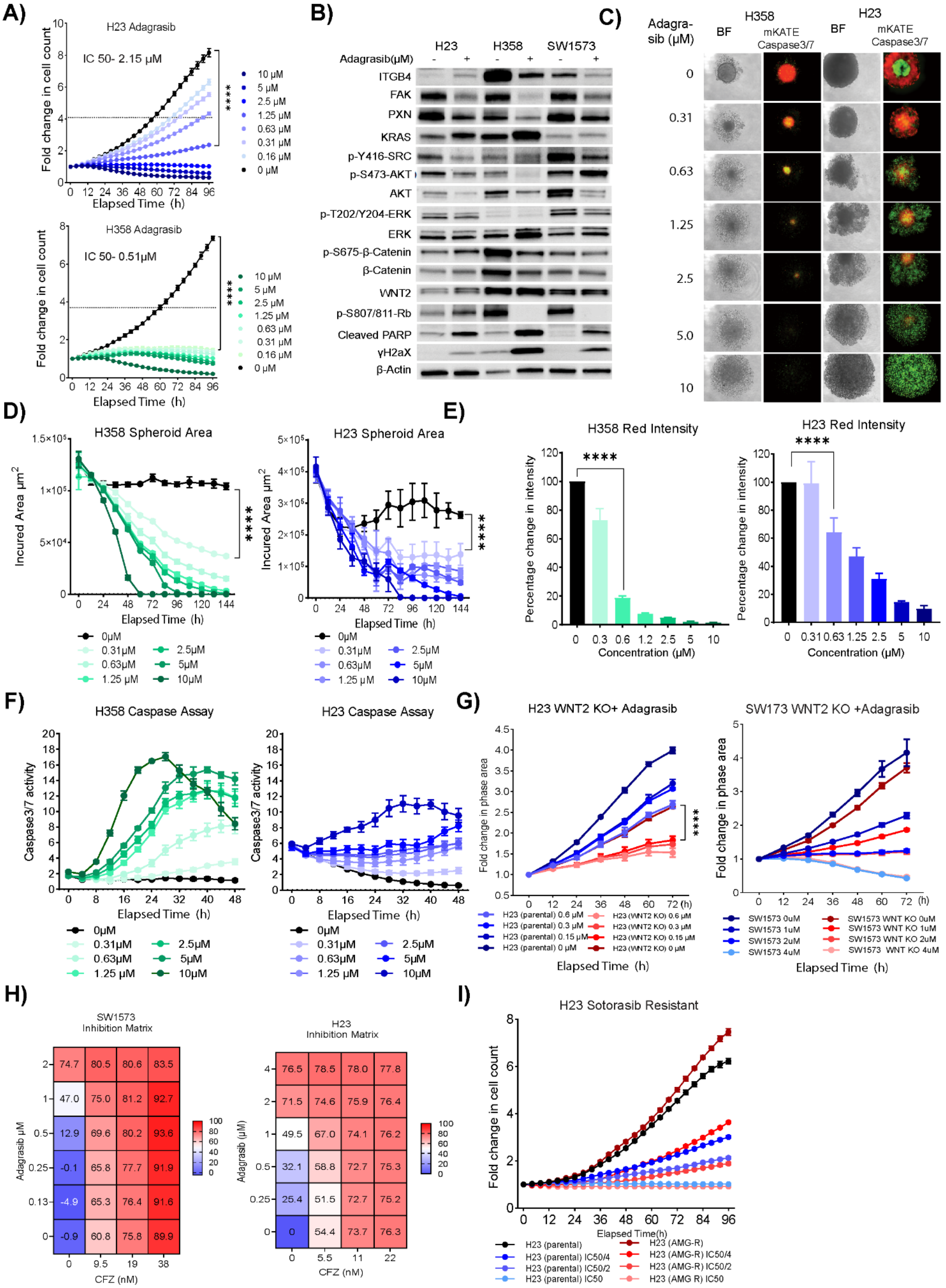

Supplementary Figure 8. Supporting data for Figure 4. **A**) H358, H23, and SW1573 cells were treated with increasing concentration (0.6-10 µM) of adagrasib (MRTX) for 72 h to determine the effect on cell proliferation. The dotted line represents a fold change in cell count which corresponds to half of the total fold change shown by control cells. Two-way ANOVA was used to calculate the statistical significance for each time point and each drug concentration, n = 3 per group, ****p<0.0001. **B**) H23, H358, and SW1573 cells were treated with adagrasib IC50 concentration for 72 h, and changes in protein expression and signaling were determined by immunoblot. **C**) Images of the H358, H23, and SW1573 cell line-derived spheroids after 144h of adagrasib treatment with an increasing concentration (0.31-10 µM). **D**) Changes in the spheroid area with respect to adagrasib treatment, **E**) changes in the spheroid red intensity, and **F**) changes in the caspase activity were determined. Ordinary one-way ANOVA was used to calculate the statistical significance, sample per group n=3, ****p<0.0001. **G**) Effect of WNT2 CRISPR knockout (KO) and addition of adagrasib on cell proliferation over the course of 96 h in H23 (left graph) and SW1573 cells (right graph). Two- way ANOVA test was used to calculate the statistical significance., sample per group, n=3, ****p< 0.0001. **H**) Matrix of 6 different concentrations of adagrasib (MRTX849) and 4 different concentrations of CFZ to determine percent of inhibition at various combinations of doses in SW1573 and H23 cells. **I)** H23 parental cells and H23 sotorasib (20 µM)-resistant cells were treated with adagrasib (IC50/4, IC50/2, and IC50) to determine fold change in cell count over 96 h.

Supplementary Figure 9

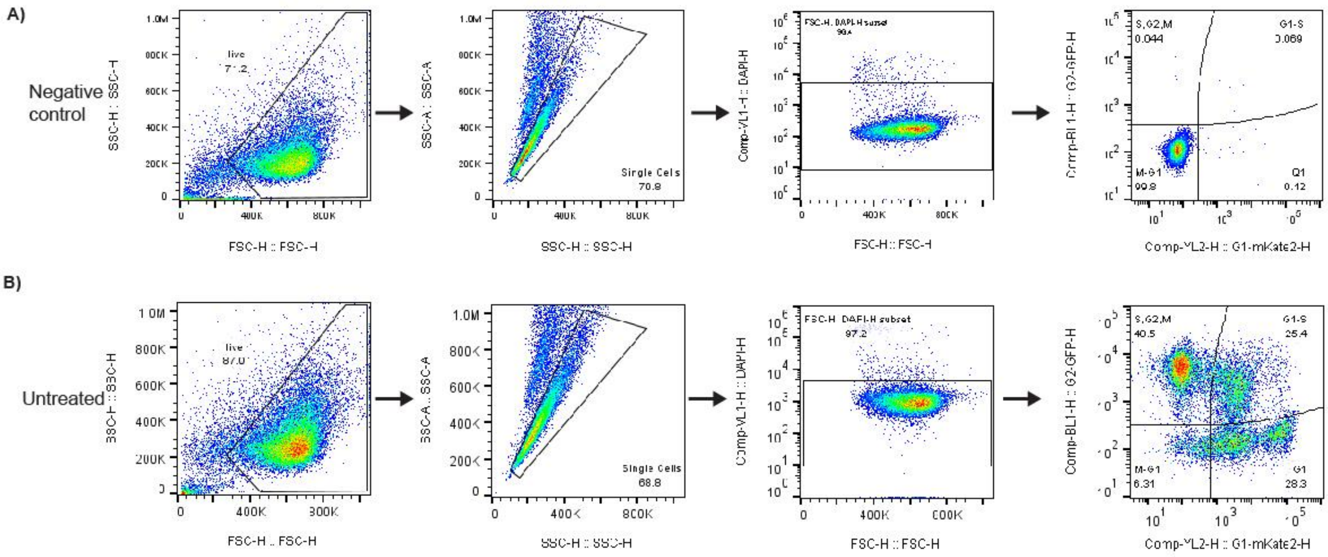

Supplementary Figure 9. Supporting data for Figure 6. Gating strategy for Cell Cycle analysis. **A**) The SW1573 regular cells were used as a control for analyzing the negative population and the back-ground fluorescence. **B**) The SW1573 untreated stable cell line expressing fluorescent markers were analyzed for singlet, and DAPI negative population in the same way as the regular cells. Next, the DAPI negative population were gated in reference to the regular cells for double negative, green, red, or both positive. The same gating strategy was used for the cells treated with drugs.

Supplementary figure 10

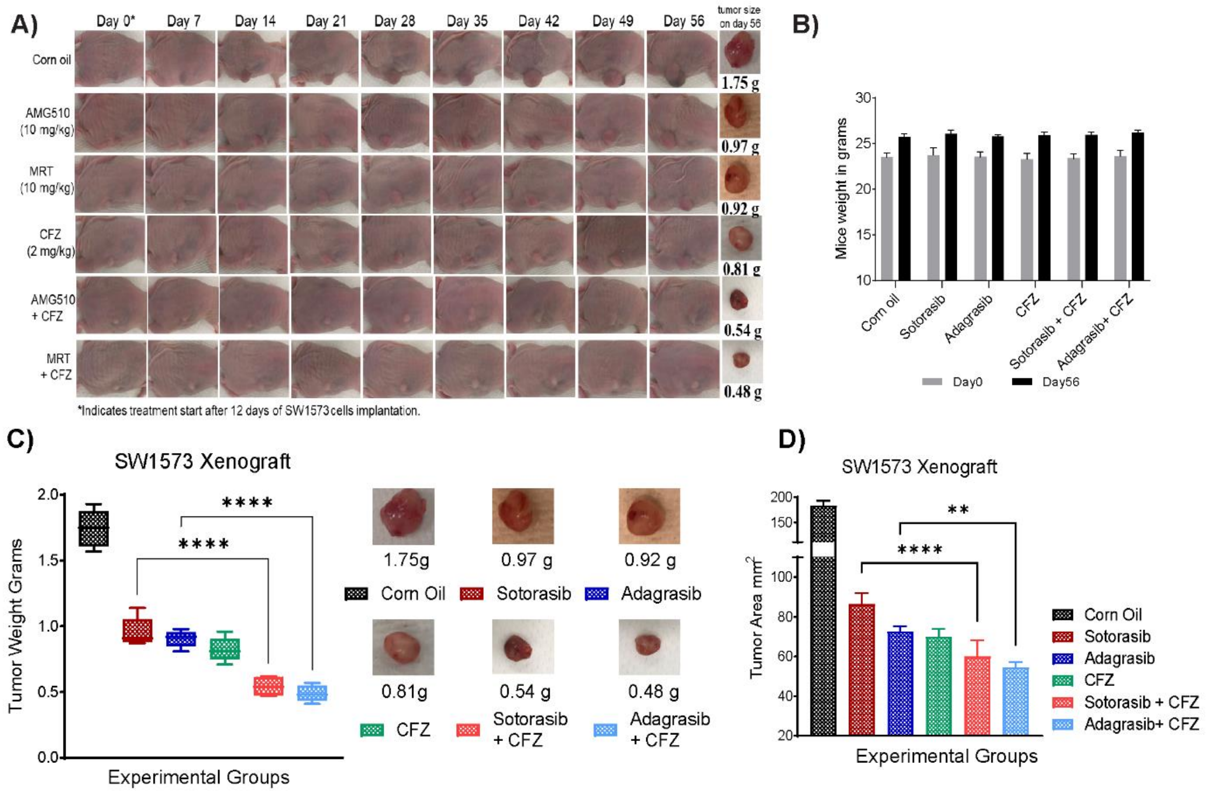

Supplementary Figure 10. Supporting data for Figure 7. **A**) Photos of mice SW1573 xenografts from day 0 to day 56 (end of the experiment) with sotorasib (AMG510) (10 mg/kg), adagrasib (MRTX849) (10 mg/kg), and CFZ (2 mg/kg) single treatments as well as drug combination treatments (sotorasib + CFZ, adagrasib + CFZ). Photos of excised tumors on day 56. **B**) No major changes in the weight (g) of mice on day 0 and day 56 (end of experiment) of treatment indicated no drug induced toxicity. **C** and **D**) Tumor weight (g) and area (mm^2) of mice SW1573 xenografts after sotorasib (AMG510) (10 mg/kg), adagrasib (MRTX849) (10 mg/kg), and CFZ (2 mg/kg) single treatments as well as drug combination treatments (sotorasib + CFZ, adagrasib + CFZ). One-way ANOVA was used for calculating statistical significance across various treatments, n= 5.

GSEA Enrichment for Top MSigDB Hallmark Gene Sets (Negative Enrichment)

Table S1: Parent vs Control

NAME	SIZE	ES	NES	NOM p-val	FDR q-val	FWER p-val	RANK AT MAX	LEADING EDGE
HALLMARK_TNFA_SIGNALING_VIA_NFKB	196	-0.62296	-1.93805	0	0	0	2070	tags=41%, list=9%, signal=45%
HALLMARK_KRAS_SIGNALING_UP	199	-0.5411	-1.71656	0	0.003865	0.003	1848	tags=30%, list=8%, signal=32%
HALLMARK_INFLAMMATORY_RESPONSE	199	-0.48078	-1.5124	0	0.040905	0.043	1708	tags=23%, list=8%, signal=25%
HALLMARK_COAGULATION	138	-0.45929	-1.42064	0.01408451	0.092133	0.124	2327	tags=25%, list=10%, signal=28%
HALLMARK_IL2_STAT5_SIGNALING	199	-0.42444	-1.33019	0.00980392	0.147592	0.228	1857	tags=23%, list=8%, signal=24%
HALLMARK_HYPOXIA	197	-0.41089	-1.30231	0.01	0.152178	0.272	2205	tags=26%, list=10%, signal=28%
HALLMARK_ESTROGEN_RESPONSE_LATE	198	-0.40885	-1.28441	0.00980392	0.152265	0.305	1536	tags=21%, list=7%, signal=22%
HALLMARK_CHOLESTEROL_HOMEOSTASIS	74	-0.41868	-1.16157	0.15315315	0.390869	0.658	1851	tags=20%, list=8%, signal=22%
HALLMARK_COMPLEMENT	199	-0.36111	-1.14793	0.09708738	0.383787	0.689	2359	tags=23%, list=11%, signal=25%
HALLMARK_ALLOGRAFT_REJECTION	197	-0.36355	-1.13531	0.1092437	0.378363	0.722	1931	tags=14%, list=9%, signal=15%
HALLMARK_IL6_JAK_STAT3_SIGNALING	85	-0.38577	-1.10947	0.17326732	0.413519	0.792	1536	tags=21%, list=7%, signal=23%
HALLMARK_UNFOLDED_PROTEIN_RESPONSE	110	-0.34266	-1.02146	0.36645964	0.724406	0.952	3348	tags=32%, list=15%, signal=37%
HALLMARK_MTORC1_SIGNALING	199	-0.31592	-1.0143	0.36036035	0.701972	0.957	3041	tags=22%, list=14%, signal=25%
HALLMARK_PANCREAS_BETA_CELLS	40	-0.39895	-1.01059	0.4067164	0.669527	0.96	437	tags=8%, list=2%, signal=8%
HALLMARK_PI3K_AKT_MTOR_SIGNALING	105	-0.33019	-0.99207	0.4702381	0.71006	0.974	1528	tags=12%, list=7%, signal=13%
HALLMARK_GLYCOLYSIS	198	-0.31375	-0.98447	0.46363637	0.695747	0.978	2194	tags=18%, list=10%, signal=20%
HALLMARK_APOPTOSIS	161	-0.3179	-0.97147	0.5862069	0.710535	0.985	2485	tags=25%, list=11%, signal=28%
HALLMARK_PROTEIN_SECRETION	95	-0.32763	-0.9632	0.55737704	0.707028	0.988	4848	tags=36%, list=22%, signal=46%
HALLMARK_UV_RESPONSE_UP	158	-0.30796	-0.94393	0.620438	0.749046	0.993	1566	tags=15%, list=7%, signal=16%
HALLMARK_ANGIOGENESIS	36	-0.37013	-0.92426	0.5669014	0.784536	0.998	1393	tags=25%, list=6%, signal=27%
HALLMARK_APICAL_SURFACE	44	-0.34394	-0.86937	0.7246377	0.909679	1	675	tags=9%, list=3%, signal=9%
HALLMARK_TGF_BETA_SIGNALING	54	-0.30018	-0.79749	0.8649789	0.980254	1	3160	tags=28%, list=14%, signal=32%
HALLMARK_REACTIVE_OXYGEN_SPECIES_PATHWAY	49	-0.28687	-0.76106	0.93189967	0.965567	1	1274	tags=10%, list=6%, signal=11%

Table S2 : Resistant vs Control

NAME	SIZE	ES	NES	NOM p-va	FDR q-val	FWER p-val	RANK AT MAX	LEADING EDGE
HALLMARK_KRAS_SIGNALING_UP	199	-0.51575	-1.53955	0	0.065328	0.062	1679	tags=28%, list=8%, signal=30%
HALLMARK_TNFA_SIGNALING_VIA_NFKB	196	-0.51549	-1.51841	0.002347	0.045646	0.088	2503	tags=38%, list=11%, signal=43%
HALLMARK_ESTROGEN_RESPONSE_LATE	198	-0.44745	-1.32908	0.02027	0.251059	0.524	1588	tags=19%, list=7%, signal=20%
HALLMARK_COAGULATION	138	-0.45693	-1.31026	0.032037	0.222351	0.594	2476	tags=25%, list=11%, signal=28%
HALLMARK_PANCREAS_BETA_CELLS	40	-0.54615	-1.30135	0.109474	0.196526	0.63	2920	tags=23%, list=13%, signal=26%
HALLMARK_INFLAMMATORY_RESPONSE	199	-0.42488	-1.24562	0.053241	0.266107	0.812	1920	tags=19%, list=9%, signal=21%
HALLMARK_HYPOXIA	197	-0.40424	-1.20423	0.070423	0.327336	0.902	2411	tags=24%, list=11%, signal=27%
HALLMARK_IL2_STAT5_SIGNALING	199	-0.39847	-1.17856	0.089806	0.356398	0.951	1966	tags=22%, list=9%, signal=24%
HALLMARK_ALLOGRAFT_REJECTION	197	-0.4017	-1.17816	0.117506	0.318021	0.952	1992	tags=14%, list=9%, signal=15%
HALLMARK_CHOLESTEROL_HOMEOSTASIS	74	-0.45228	-1.17184	0.182844	0.300496	0.958	3028	tags=28%, list=14%, signal=33%
HALLMARK_COMPLEMENT	199	-0.37411	-1.11629	0.192857	0.417416	0.989	2393	tags=20%, list=11%, signal=22%
HALLMARK_ESTROGEN_RESPONSE_EARLY	198	-0.36894	-1.08978	0.224638	0.457896	0.997	1695	tags=20%, list=8%, signal=21%
HALLMARK_BILE_ACID_METABOLISM	112	-0.39142	-1.0816	0.265734	0.447419	0.997	2966	tags=18%, list=13%, signal=21%
HALLMARK_APOPTOSIS	161	-0.35637	-1.03046	0.35049	0.576587	1	2449	tags=22%, list=11%, signal=25%
HALLMARK_IL6_JAK_STAT3_SIGNALING	85	-0.3834	-1.02639	0.402273	0.551077	1	2467	tags=25%, list=11%, signal=28%
HALLMARK_PROTEIN_SECRETION	95	-0.36738	-1.00253	0.455172	0.593334	1	5600	tags=33%, list=25%, signal=43%
HALLMARK_GLYCOLYSIS	198	-0.3281	-0.97203	0.527273	0.664447	1	2170	tags=14%, list=10%, signal=16%
HALLMARK_APICAL_SURFACE	44	-0.40272	-0.95174	0.535792	0.695318	1	1920	tags=18%, list=9%, signal=20%
HALLMARK_PI3K_AKT_MTOR_SIGNALING	105	-0.32589	-0.90711	0.676405	0.817614	1	2026	tags=10%, list=9%, signal=11%
HALLMARK_HEME_METABOLISM	195	-0.30426	-0.90178	0.755196	0.793542	1	2994	tags=17%, list=13%, signal=19%
HALLMARK_REACTIVE_OXYGEN_SPECIES_PATHWA	49	-0.3173	-0.78131	0.872	1	1	1978	tags=12%, list=9%, signal=13%
HALLMARK_MTORC1_SIGNALING	199	-0.26135	-0.77537	0.993319	0.960994	1	3415	tags=18%, list=15%, signal=21%

GSEA Enrichment for Top MSigDB Hallmark Gene Sets (Positive Enrichment)

Table S3: Parent vs Control

NAME	SIZE	ES	NES	NOM p-va	FDR q-val	FWER p-val	RANK AT MAX	LEADING EDGE
HALLMARK_NOTCH_SIGNALING	32	0.673394	1.411344	0.042735	0.330861	0.392	1390	tags=28%, list=6%, signal=30%
HALLMARK_E2F_TARGETS	200	0.521423	1.35996	0.011211	0.322734	0.635	8001	tags=69%, list=36%, signal=107[illegible]
HALLMARK_KRAS_SIGNALING_DN	197	0.487645	1.269073	0.049217	0.617134	0.954	3964	tags=31%, list=18%, signal=38%
HALLMARK_MYOGENESIS	200	0.475538	1.242037	0.067873	0.597416	0.976	3358	tags=24%, list=15%, signal=27%
HALLMARK_OXIDATIVE_PHOSPHORYLATION	185	0.463598	1.201503	0.105499	0.692708	0.999	7757	tags=54%, list=35%, signal=82%
HALLMARK_APICAL_JUNCTION	199	0.459913	1.195335	0.115813	0.611523	1	3535	tags=24%, list=16%, signal=28%
HALLMARK_FATTY_ACID_METABOLISM	158	0.458687	1.168819	0.16916	0.648559	1	6769	tags=43%, list=30%, signal=61%
HALLMARK_G2M_CHECKPOINT	196	0.443775	1.159465	0.146667	0.611812	1	8122	tags=65%, list=37%, signal=102[illegible]
HALLMARK_ADIPOGENESIS	199	0.441777	1.156571	0.164179	0.555881	1	5778	tags=38%, list=26%, signal=50%
HALLMARK_INTERFERON_GAMMA_RESPONSE	199	0.432797	1.144417	0.170455	0.550286	1	4378	tags=34%, list=20%, signal=42%
HALLMARK_ANDROGEN_RESPONSE	98	0.453039	1.12301	0.264743	0.584235	1	1799	tags=14%, list=8%, signal=15%
HALLMARK_WNT_BETA_CATENIN_SIGNALING	42	0.503585	1.108841	0.306343	0.590028	1	4164	tags=36%, list=19%, signal=44%
HALLMARK_MYC_TARGETS_V1	198	0.421842	1.10217	0.252809	0.56975	1	9220	tags=68%, list=42%, signal=115[illegible]
HALLMARK_HEDGEHOG_SIGNALING	36	0.509639	1.096819	0.345021	0.545594	1	2937	tags=39%, list=13%, signal=45%
HALLMARK_PEROXISOME	104	0.439267	1.079564	0.339853	0.566194	1	5234	tags=28%, list=24%, signal=36%
HALLMARK_UV_RESPONSE_DN	140	0.414754	1.05038	0.381008	0.625306	1	2616	tags=24%, list=12%, signal=27%
HALLMARK_ESTROGEN_RESPONSE_EARLY	198	0.386747	1.005851	0.452915	0.741449	1	1883	tags=20%, list=8%, signal=21%
HALLMARK_BILE_ACID_METABOLISM	112	0.391066	0.978972	0.506555	0.787666	1	2709	tags=17%, list=12%, signal=19%
HALLMARK_EPITHELIAL_MESENCHYMAL_TRANSITION	197	0.371756	0.970656	0.541806	0.771225	1	1382	tags=18%, list=6%, signal=19%
HALLMARK_MITOTIC_SPINDLE	198	0.367024	0.967693	0.559511	0.741681	1	6440	tags=41%, list=29%, signal=58%
HALLMARK_DNA_REPAIR	148	0.377362	0.963988	0.565865	0.716561	1	8178	tags=50%, list=37%, signal=79%
HALLMARK_INTERFERON_ALPHA_RESPONSE	95	0.389046	0.947411	0.589176	0.727425	1	3849	tags=37%, list=17%, signal=44%
HALLMARK_SPERMATOGENESIS	135	0.347525	0.878734	0.734503	0.865612	1	1493	tags=9%, list=7%, signal=9%
HALLMARK_XENOBIOTIC_METABOLISM	200	0.333968	0.873597	0.760405	0.840551	1	3867	tags=25%, list=17%, signal=30%
HALLMARK_P53_PATHWAY	199	0.327129	0.859227	0.804077	0.836609	1	3691	tags=29%, list=17%, signal=35%
HALLMARK_HEME_METABOLISM	195	0.293298	0.76795	0.959596	0.958063	1	4644	tags=26%, list=21%, signal=33%
HALLMARK_MYC_TARGETS_V2	58	0.278003	0.644872	0.983007	0.993927	1	7419	tags=41%, list=33%, signal=62%

Table S4: Resistant vs Control

NAME	SIZE	ES	NES	NOM p-val	FDR q-val	FWER p-val	RANK AT MAX	LEADING EDGE
HALLMARK_E2F_TARGETS	200	0.541302	1.569499	0	0.053316	0.052	7154	tags=69%, list=32%, signal=100[illegible]
HALLMARK_MYC_TARGETS_V1	198	0.513811	1.484508	0	0.080932	0.144	8196	tags=70%, list=37%, signal=110[illegible]
HALLMARK_NOTCH_SIGNALING	32	0.645553	1.422721	0.0405904	0.1179	0.293	1385	tags=28%, list=6%, signal=30%
HALLMARK_INTERFERON_GAMMA_RESPONSE	199	0.471933	1.372172	0.0092251	0.161171	0.475	3104	tags=32%, list=14%, signal=37%
HALLMARK_MYOGENESIS	200	0.466444	1.347297	0.013913	0.166194	0.562	1338	tags=14%, list=6%, signal=15%
HALLMARK_OXIDATIVE_PHOSPHORYLATION	185	0.457817	1.315897	0.0182724	0.187481	0.68	7282	tags=55%, list=33%, signal=81%
HALLMARK_INTERFERON_ALPHA_RESPONSE	95	0.476453	1.256513	0.0868794	0.279095	0.86	3197	tags=41%, list=14%, signal=48%
HALLMARK_HEDGEHOG_SIGNALING	36	0.531619	1.200462	0.1809524	0.398196	0.968	1257	tags=25%, list=6%, signal=26%
HALLMARK_G2M_CHECKPOINT	196	0.397721	1.145861	0.1523973	0.548899	0.996	6526	tags=54%, list=29%, signal=75%
HALLMARK_ADIPOGENESIS	199	0.394094	1.144881	0.119171	0.497034	0.996	3131	tags=18%, list=14%, signal=20%
HALLMARK_APICAL_JUNCTION	199	0.379555	1.089928	0.2512479	0.661549	1	2827	tags=22%, list=13%, signal=25%
HALLMARK_EPITHELIAL_MESENCHYMAL_TRANSITION	197	0.378403	1.089751	0.2366149	0.607475	1	1715	tags=22%, list=8%, signal=23%
HALLMARK_KRAS_SIGNALING_DN	197	0.378758	1.085593	0.2358003	0.577376	1	1931	tags=18%, list=9%, signal=19%
HALLMARK_UV_RESPONSE_DN	140	0.391546	1.081732	0.2733686	0.550272	1	2700	tags=24%, list=12%, signal=27%
HALLMARK_ANDROGEN_RESPONSE	98	0.393654	1.051471	0.3409091	0.616522	1	2125	tags=16%, list=10%, signal=18%
HALLMARK_MITOTIC_SPINDLE	198	0.361987	1.049465	0.327957	0.584321	1	6354	tags=45%, list=29%, signal=62%
HALLMARK_FATTY_ACID_METABOLISM	158	0.370456	1.030662	0.3592401	0.617055	1	5781	tags=37%, list=26%, signal=50%
HALLMARK_MYC_TARGETS_V2	58	0.402328	0.987814	0.4745763	0.740444	1	7107	tags=62%, list=32%, signal=91%
HALLMARK_TGF_BETA_SIGNALING	54	0.394073	0.949854	0.5454546	0.845907	1	1953	tags=13%, list=9%, signal=14%
HALLMARK_XENOBIOTIC_METABOLISM	200	0.330547	0.944357	0.6	0.824172	1	2364	tags=18%, list=11%, signal=20%
HALLMARK_PEROXISOME	104	0.354794	0.941124	0.5996441	0.796802	1	3964	tags=25%, list=18%, signal=30%
HALLMARK_P53_PATHWAY	199	0.325501	0.936355	0.6238698	0.776121	1	2452	tags=21%, list=11%, signal=24%
HALLMARK_DNA_REPAIR	148	0.316832	0.895441	0.7453626	0.879671	1	7093	tags=45%, list=32%, signal=65%
HALLMARK_WNT_BETA_CATENIN_SIGNALING	42	0.380664	0.885431	0.6672828	0.875257	1	3507	tags=36%, list=16%, signal=42%
HALLMARK_UV_RESPONSE_UP	158	0.311395	0.882774	0.7743119	0.848028	1	3061	tags=24%, list=14%, signal=28%
HALLMARK_ANGIOGENESIS	36	0.37646	0.85193	0.6994434	0.900251	1	806	tags=17%, list=4%, signal=17%
HALLMARK_SPERMATOGENESIS	135	0.278299	0.77529	0.9658703	0.991563	1	3142	tags=14%, list=14%, signal=16%
HALLMARK_UNFOLDED_PROTEIN_RESPONSE	110	0.250156	0.675683	0.996633	0.992828	1	619	tags=3%, list=3%, signal=3%
